# Rapid inducible protein displacement in *Plasmodium* *in vivo* and *in vitro *using knocksideways technology

**DOI:** 10.12688/wellcomeopenres.11005.1

**Published:** 2017-03-14

**Authors:** Katie R. Hughes, Andy P. Waters

**Affiliations:** 1 Wellcome Centre for Molecular Parasitology, Institute of Infection, Immunity and Inflammation, College of Medical Veterinary & Life Sciences, University of Glasgow, Glasgow, G12 8TA, UK

**Keywords:** Plasmodium berghei, Inducible Technology, Knocksideways, Malaria, GAP50, GFP, Transmission

## Abstract

A deeper understanding of the biology of the *Plasmodium* parasite is essential in order to identify targets for interventions, with the ultimate aim of eliminating malaria. Determining the function(s) of essential proteins in *Plasmodium* has, until recently, been hampered by the lack of efficient conditional systems to abrogate proteins. We report the adaptation of a conditional technology, knocksideways (KS), for use in *Plasmodium berghei, *which can potentially rapidly inactivate proteins of interest through relocalisation. The system is induced using rapamycin, which allows for KS both *in vitro *and *in vivo *and is effective more rapidly than any other reported system. KS utilises pairs of fluorescent tags that facilitate live imaging and allows for rapid confirmation of efficient protein redistribution on live parasites, allowing for streamlined workflows. We demonstrate the characteristics of the system using transgenically expressed cytoplasmic GFP and provide proof of principle by inducibly redistributing a number of proteins with different native, subcellular locations.  We also demonstrate that KS can be applied to both mammalian and insect stages of *Plasmodium*. KS expands the range of (conditional) technologies for genetic manipulation of malaria parasites and offers the potential to be further developed for medium throughput phenotype screens.

## Introduction

*Plasmodium* parasites are transmitted between hosts by a mosquito vector and display many unique biological features. Despite the wealth of genomic, transcriptomic, proteomic and metabolomic data about *Plasmodium*, an understanding is still lacking of the functions of the protein products of ~1939 parasite genes that remain annotated as a conserved protein of unknown function ( Bozdech *et al.*, 2003; Gardner *et al.*, 2002; Hall *et al.*, 2005; Khan *et al.*, 2005; Otto *et al.*, 2014; Rutledge *et al.*, 2017) ( http://plasmodb.org/plasmo/). Their study and assignation of function to such proteins remains a significant barrier to a full understanding and exploitation of the biology of these haploid parasites in the search for therapies, as essential genes cannot be studied by traditional gene disruption in the pathogenic asexual blood stage forms. There are numerous conditional technologies now adapted for use in apicomplexan parasites, which are, in principle, available to investigate the function(s) of these essential genes. However, most suffer from an unpredictable lag-phase and efficacy that is dependent upon a number of considerations, including the efficiency of induced endonuclease or protein degradation activities, the abundance of the target protein and its natural half-life ( de Koning-Ward *et al.*, 2015). Lastly, all approaches to genetic manipulation of a haploid genome are likely to be applicable to only a variable proportion of that genome and so the availability of a plurality of approaches is highly desirable.

**Figure 1.**
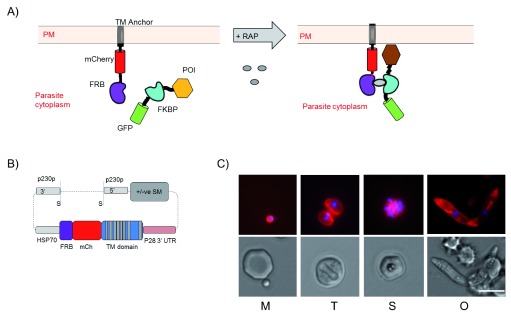
The principle of knocksideways. ( **A**) Schematic of principle of knocksideways. FRB::mCherry fusion is anchored into the cell plasma membrane via a multi-pass transmembrane domain (TM). The protein of interest (POI) is tagged with FKBP::GFP fusion. Addition of rapamycin (RAP) stimulates heterodimerisation of FRB and FKBP and the POI is relocalised to the membrane with possible phenotypic consequences (PM, plasma membrane). ( **B**) Schematic of fusion construct to generate **K**nock **S**ideways **P**arental line (KSP2). See also experimental procedures and Figure S1. (S indicates Sac II sites used to linearise vector). ( **C**) Images of KSP2 showing membrane localised mCherry and Hoechst stain (top) and DIC (lower) for parasite stages merozoite (m), trophozoite (t), schizont (s) and ookinete (o). All observed parasites showed similar mCherry expression. Representative images shown of >10 parasites observed on >3 independent occasions. Scale bar = 5 µm.

Inducible protein displacement technologies represent approaches to phenotype assessment that have not yet been applied to Apicomplexans and offer some significant advantages to currently available approaches ( Geda *et al.*, 2008; Haruki *et al.*, 2008; Robinson *et al.*, 2010). One format knocksideways (KS) typically acts on a time scale of seconds ( Robinson *et al.*, 2010) and generates inducible protein inactivation by subcellular relocalisation. The addition of rapamycin (rap) induces heterodimerisation of two rapamycin-binding protein domains, FKBP and FRB, which are used to tag the Protein of Interest (POI) and serve as a (membrane-localised) sink for displaced proteins, respectively ( Figure 1A) ( Robinson *et al.*, 2010). KS has previously been developed for the study of clathrin-coated vesicle mediated protein trafficking in mammalian cells, and in yeast similar systems have been used to study Golgi formation and nuclear protein function ( Haruki *et al.*, 2008; Papanikou *et al.*, 2015; Robinson *et al.*, 2010). This demonstrates that for a broad range of proteins relocalisation may be an effective way to inactivate the protein in order to study function.

We have developed KS in *P. berghei* to be applicable in principle to a wide variety of proteins, and which combines the relocalising domains with fluorescent markers (GFP and mCherry fused to the anchor FRB and the POI FKBP, respectively) to enable rapid proof of relocalisation through microscopy and visual phenotyping assays. In order to demonstrate the system for use in *Plasmodium* spp., we use a FKBP::GFP transgene, with which we thoroughly demonstrate that protein relocalisation is effective in all life cycle stages. We then choose a wide range of *Plasmodium* proteins and, by adding a c-terminal FKBP::GFP tag to each, we perform a preliminary analysis on each of these to show that relocalisation is effective on a range of parasite proteins with different subcellular localisations. Through a growth screen, we identify two proteins where we see statistically significant growth defects after induction of relocalisation.

## Results

### Generation of parental lines in *P. berghei*

A parental line for KS was generated to express a high level of FRB anchor protein localised to a defined, but accessible, subcellular localisation in *P. berghei*. The FRB domain was fused to a transmembrane (TM) protein (PBANKA_110790), which is the *P. berghei* homologue of triose phosphate transporter protein PfoTPT, an apicoplast outer membrane protein predicted to have 10 TM domains with both N- and C-termini cytoplasmically exposed ( Mullin *et al.*, 2006). mCherry ( Graewe *et al.*, 2009) was also included generating the fusion protein FRB::mCherry::TM. High expression was achieved using 1.7 kbp of HSP70 promoter (PBANKA_0711190) and the construct was integrated into the 230p locus to generate the KS parental line ( Figure 1B). Two similar parental lines were generated, **K**nock **S**ideways **P**arental **1** (KSP1), in which the FRB::mCherry::TM protein regulated with a P45/48 stable 3’ UTR, and KSP2 with the FRB::mCherry::TM regulated by a P28 3’ UTR ( Figure S1A). KSP2 gave approximately 50% reduced expression of mCherry signal, but resulted better conversion rate to ookinetes ( Figures S1C and D). Preliminary experiments carried out in both backgrounds gave identical results ( Figure S2). The fusion protein predominantly localised to the parasite plasma membrane in live parasites, including defining the central cavity ( Grüring *et al.*, 2011). Closer examination revealed some expression on intracellular membranous structures, including a structure morphologically consistent with the apicoplast as predicted by the native localisation of the original PboTPT TM domain protein anchor ( Mullin *et al.*, 2006) ( Figure 1C; Figure S1F). The membrane localisation is conferred by the PboTPT domain, as a line expressing FRB::mCherry showed mCherry throughout the cytoplasm ( Figure S1E). Expression of FRB::mCherry::TM appeared to have no detrimental effect on asexual growth ( Figure S4B) and expression was stable over several passages and through mosquito transmission.

### KS functions as a highly efficient protein translocation system

**Figure 2.**
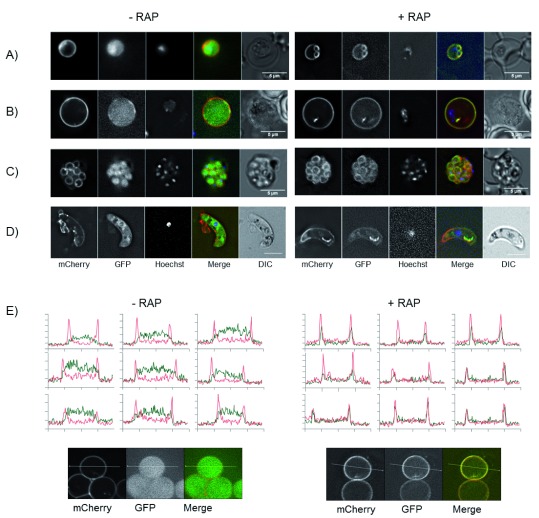
Knocksideways relocalisation of GFP. ( **A**– **D**) Images of GFP _KSP2_ parasites. Trophozoite ( **A**), gametocyte ( **B**) schizont ( **C**) and ookinete ( **D**) without (left) or with (right) 200 nM rapamycin (RAP) *in vitro*, scale bar =5 µm. All observed parasites showed similar GFP distribution. Images representative of >10 parasites for each stage observed on 3 independent occasions. See also Figure S2. ( **E**) Line profiles of a 10 µm slice through individual parasites showing mCherry (red) and GFP (green) minus and plus 200 nm RAP. Line profiles representative of 11 parasites analysed. Line=10 µm.

**Figure 3.**
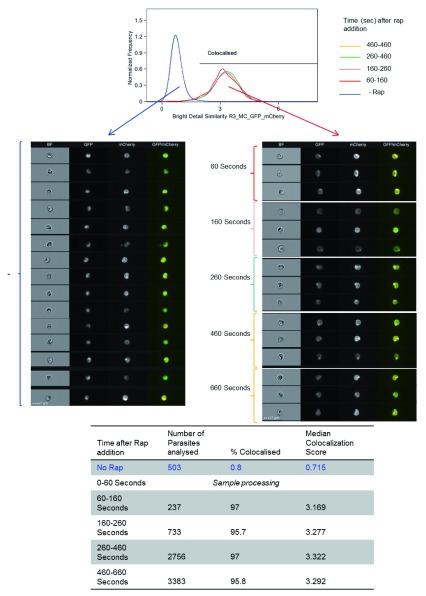
Knocksideways relocalisation of GFP occurs very rapidly. Imaging flow cytometry analysis of parasites. A histogram is presented of colocalisation between GFP and mCherry for time defined segments of parasites acquired at various times, as indicated after rapamycin (Rap) addition. Untreated parasites were acquired in a separate sample and files merged for analysis. The colocalisation feature wizard from IDEAS ^TM^ (Amnis-Merck) software was used and bright intensity colocalisation score for each image gated on mCherry and GFP positive and mCherry focussed images was calculated. In addition, a gallery of images is shown, which was obtained from ImageStream ^X®^ showing untreated parasites (left) or 60–160 seconds after 200 nM Rap addition (right). Scale bar = 7 µm. The table shows the number of parasites included in the analysis, % colocalisation and median colocalisation score for each treatment.

The ability of KS to relocalise protein was tested with GFP. Parental line KSP2 was transfected with a construct to express FKBP::GFP; after positive selection, parasites were FACS sorted based on GFP and mCherry expression to generate an isogenic line GFP _KSP2_. Live parasites were observed by fluorescence microscopy and addition of rapamycin (RAP) that caused GFP, which was diffusely localised throughout the parasite cytoplasm, to re-localise to the mCherry labelled parasite plasma membrane. This observation was uniform across all parasites in all blood stages from rings to mature schizonts and merozoites, as well as sexual stage gametocytes and ookinetes ( Figure 2). Relocalisation was observed by microscopy after addition of 1.6–1000 nM RAP ( Figure S2D). For subsequent *in vitro* experiments, a concentration of 200 nM was used, which had no effect on asexual or sexual stage parasite development. Relocalisation was dependent on the presence of both the FRB and FKBP domains as a line expressing FKBP::GFP in a WT background (GFP _KSWT_) showed no relocalisation of GFP on addition of RAP ( Figure S5A). Additionally, the membrane localisation of the mCherry::FRB was unaffected by the addition of RAP ( Figure S5B). The FKBP::GFP relocalisation experiments gave identical results in independent lines in both parental lines KSP1 and KSP2 ( Figure S2 and Figure S3). Relocalisation of GFP could be observed by microscopy as early as three minutes after addition of RAP, a temporal resolution only limited by handling speeds ( Figure S2E). However, a better measure of the speed and extent of relocalisation could be obtained using imaging flow cytometry technology (IFC). This allows for imaging and analysis of hundreds of cells per second. Magnetically enriched GFP _KSP2_ parasites were treated by addition of RAP (200 nM) and immediately placed on an ImageStream ^®X^ mk II cytometer. Acquisition of events initiated approximately one minute after addition of RAP and was continued for 10 minutes. Complete relocalisation was observed in the first parasites observed, as measured by the colocalisation of GFP and mCherry, and there was no further increase in colocalisation over the entire 10 minutes ( Figure 3; see also Supplementary methods and Figure S7). We could also show using IFC colocalisation that GFP relocalisation was efficient after a 20 minute incubation in concentrations down to 2 nM ( Figure S6).

### KS functions effectively *in vivo*

**Figure 4.**
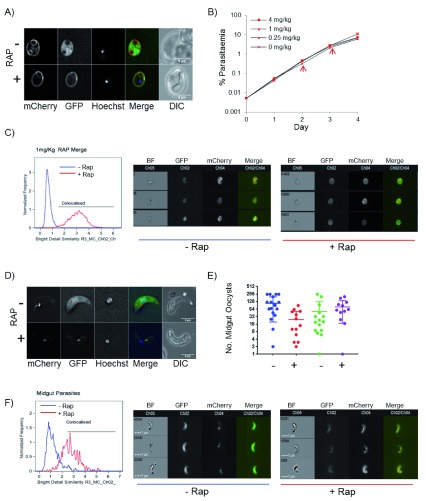
Knocksideways *in vivo*. ( **A**) Relocalisation of GFP after rapamycin (RAP) induction *in vivo*. Images show parasites from drops of blood taken 20 minutes after IP injection of 1 mg/ml. Scale bar = 5 µm. ( **B**) Growth of parasites is unaffected by RAP-induced relocalisation of GFP. Eight mice were inoculated at 0.005% parasitaemia with GFP _KS2_ parasites and induced with RAP at 0 – 4 mg/kg, as indicated by IP injection on days 2 and 3 (arrow). (N=2). Parasitaemia was measured daily by flow cytometry gating on mCherry and Hoechst stain. ( **C**) Histogram of colocalisation measured by imaging flow cytometry (IFC) on an ImageStream ^X^ MkII cytometer showing minus RAP (blue) or 2 hours after treatment with 1 mg/kg RAP (red). Right panel shows a gallery of images of parasites minus (left) or plus rapamycin (right). Scale bar = 7 µm. The analysis totals 4917 parasites minus rapamycin and 2716 parasites plus RAP. ( **D**) Images of ookinetes isolated from mosquito midguts 24 hours after feeding on GFP _KSP2_ infected mice. Mice were treated with 1 mg/kg RAP 1 hour prior to the mosquito feed. ( **E**) Number of midgut oocysts counted on days 10 – 14 in mosquitoes fed on untreated (-) or RAP-treated mice (+). Two independent transmission experiments shown. ( **F**) Histogram of colocalisation measured by (IFC) of parasites isolated from the mosquito midgut 24 hours after being fed on a GFP _KSP2_ infected mice treated without or with 1 mg/kg RAP 1 hour prior to mosquito feed. Galleries of images are representative images from the IFC analysis showing ookinetes without (left) or with (right) RAP. Scale bar = 7 µm. The analysis totals 560 parasites minus RAP and 366 parasites plus RAP.

KS works efficiently *in vivo*. Mice infected with GFP _KSP2_ were treated with 1 mg/kg RAP and blood stage parasites were analysed by fluorescence microscopy. Samples taken after >20 minutes showed relocalisation of GFP, as shown by IFC and fluorescent microscopy ( Figures 4A and C), and this could be achieved with RAP doses as low as 0.25 mg/kg. Importantly, no detrimental effect to the parasites was observed after addition of rapamycin. No significant difference in growth rate was detected of GFP _KSP2_ lines treated with up to 4 mg/kg RAP ( Figure 4B). RAP treatment did not affect growth of parental lines, or a line expressing FKBP::GFP in a WT background ( Figures S5A and B). RAP is rapidly metabolised, and we observed that 24h after a single dose of RAP newly invaded parasites did not have re-localised GFP; however, GFP was re-localised in 24h old gametocytes from the same sample ( Figure S3A). Further addition of RAP relocalised GFP in newly invaded parasites, showing that these were not refractory to relocalisation ( Figure S3B).

To test relocalisation in transmission stages *in vivo*, mice infected with GFP _KSP2_ were treated with RAP 1 hour prior to feeding to 250 female *Anopheles* mosquitoes. 24 hours later midguts were dissected and analysis of ookinetes revealed that GFP relocalisation was complete in parasites isolated from mosquitoes that had been fed on a RAP-treated mouse ( Figures 4D and F). There was no significant difference in the number of midgut oocysts between those exposed to RAP and the untreated parasites ( Figure 4E). No relocalisation of GFP was apparent in oocysts observed from day 6 ( Figure S4), suggesting that prolonged GFP relocalisation following gametocyte exposure to RAP does not occur beyond the gamete to ookinete transition. GFP _KSP2_ parasites transmitted back to recipient mice with similar kinetics regardless of RAP treatment of parasites in the donor mouse. Transmitted lines maintained mCherry and GFP expression and remained sensitive to RAP-induced relocalisation of GFP ( Figure S5C). Thus, the KS technology results in the very rapid and stable relocalisation of protein both *in vitro* and *in vivo,* and relocalisation of a non-essential protein to the membrane localised anchor does not have any detrimental effect on parasite growth or development.

### KS of *P. berghei* proteins

**Figure 5.**
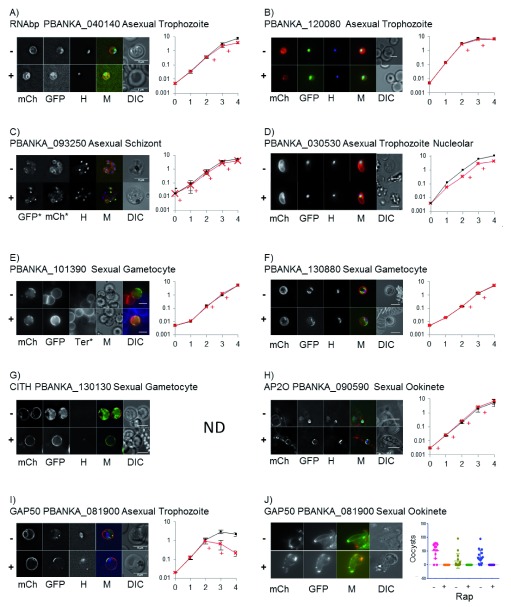
Knocksideways (KS) of *Plasmodium* proteins. ( **A**– **I**) Images of KS parasites expressing FKBP::GFP tagged protein, as indicated, showing upper panel minus (-) and lower panel plus 200 nM rapamycin (RAP) (+). Images were taken on a Deltavision core microscope and deconvoluted using softWoRx software. Showing mCherry (mCH), GFP, Hoechst ( **H**) and DIC images. Scale bars are 5 µm. Parasites in ( **E**) were stained with the RBC membrane marker Ter119 instead of Hoechst and parasites in ( **F**) show a maximum projection of the deconvoluted Z stack. Graphs show growth curves for each KS line minus (dark line) and plus (red line) Rap treatment at 1 mg/kg by IP injection usually on days 2 and 3 (+), except for ( **C** and **H**) when Rap was additionally given on day 0 and 1. Graphs show % parasitaemia (y axis) measured by flow cytometry gated on mCherry and Hoechst positive parasites over 5 days (x axis). Mean +/- SD of 2–4 mice per group. The possible growth discrepancy in ( **D**) was not significant. ( **J**) GAP50 KS ookinetes after RAP treatment for 24 hours. Plot shows the number of midgut oocyst counts on day 10–14 after *in vivo*, GAP50 KS induction one hour prior to transmission feed for three independent transmissions minus (-) and plus (+) RAP.

We tested the KS system on *P. berghei* proteins by selecting a broad range of proteins at different expression levels, patterns and physical distribution within the parasite in order to determine whether relocalisation of native proteins was possible. A plasmid construct was generated in order to introduce a C-terminal FKBP::GFP tag onto a protein of interest (POI) by single crossover homologous recombination. A region of homology for the POI was inserted into this construct into a multiple cloning site present upstream of the FKBP::GFP coding sequence. Following linearisation of the construct within this region of homology, the purified DNA was transfected into the KSP2 parental line, and after <1 week of pyrimethamine selection parasites containing the FKBP::GFP fusion at the endogenous locus were obtained. Relocalisation experiments were carried out on small drops of blood containing transfected parasites, and relocalisation was visualised by microscopy after addition of 200 nM RAP for 15 – 60 minutes ( Figure 5). In most cases, we could see relocalisation of protein as compared to the untreated parasites of the same line. Similar to the FKBP::GFP control line, relocalisation appeared to be complete and occurred in all visualised parasites.

Relocalisation could be seen of two proteins containing RNA binding domains PBANKA_040140 and PBANKA_120080. In the absence of RAP, PBANKA_040140 localised to a region surrounding the nucleus consistent with the identification of its orthologue in *P. falciparum* (PF3D7_0302800) as a nuclear protein ( Oehring *et al.*, 2012). Following RAP treatment, PBANKA_040140 colocalised with the mCherry signal at the cell membrane ( Figure 5A). PBANKA_120080 appeared to have some nuclear localisation and interestingly on RAP addition appeared to localise to a region surrounding the nucleus, as well as the cell periphery ( Figure 5B), suggesting that the FRB::mCherry::TM anchor protein was also localised to the nuclear membrane. Indeed the nuclear periphery was observed as a relocalisation destination for other proteins, most notably in sexual stage ookinetes where the AP2-O transcription factor relocalised to presumably the nuclear membrane ( Figure 5H). PBANKA_093250 was localised to punctate foci in mature schizont stage parasites and on addition of RAP can be seen to be relocalised to the periphery of the developing merozoites, showing that KS relocalisation of protein can be seen in mature schizonts ( Figure 5C). The predicted nucleolar protein PBANKA_030530 showed the expected nuclear localisation, although on addition of RAP we were unable to determine whether relocalisation had occurred ( Figure 5D). This may reflect the limiting resolution of wide field fluorescent microscopy, or that this subnuclear localisation of the protein is refractory to the KS technology.

Several known and unknown sexual stage proteins were also analysed to determine whether KS is functional on proteins expressed in these life cycle stages. PBANKA_101390 is highly expressed and appeared to localise to the periphery of mature gametocytes in uninduced parasites ( Figure 5E). Gametocytes were activated and the host red blood cell stain Ter119 was used to show that the protein was associated with the parasite and not the host red blood cell ( Figure S8A). Due to the location of the protein, we again could not resolve for this protein whether relocalisation had occurred by wide field fluorescence microscopy.

For two sexual specific proteins, we could very clearly see relocalisation. PBANKA_130880 is a protein of unknown function that had a distinct and fluid localisation within gametocytes. On addition of RAP it relocalised to the mCherry labelled membrane of the surface of the parasite ( Figure 5F). In sexual stages particularly, we could often see relocalisation of proteins to the mCherry labelled intracellular structure, which is morphologically consistent with being the apicoplast ( Figure 5F). One of the highest expressed proteins that we tested was the previously studied RNA binding protein CITH, PBANKA_130130 ( Mair *et al.*, 2010). This showed a cytoplasmic localisation in gametocytes and a very clear relocalisation after RAP addition ( Figure 5G). This shows that the area of the FRB anchor is sufficient to sequester a large amount of protein. Relocalisation of CITH was also observed in ookinete stages after addition of RAP to gametocytes ( Figure S8B). Lastly, GAP50 (PBANKA_081900) is expressed highly in both sexual and asexual stages. In late stage trophozoites when GAP50 was first expressed it localised to punctate intracellular structures and on addition of RAP relocalised to the parasite membrane ( Figure 5I).

### Growth phenotyping assays

Parasite lines that had significant asexual GFP expression were then FACS sorted to obtain isogenic populations, and *in vivo* growth curves carried out to determine whether relocalisation of that protein had an effect on parasite growth. Pairs of mice were infected at a starting parasitaemia of ~ 0.005% and infection was monitored daily by flow cytometry. In most experiments, when parasitaemia had reached 0.2 – 0.6% (day 2) mice were treated with RAP by IP injection at 1 mg/kg (+ RAP), or 3% DMSO (vehicle) to control mice (-RAP) for two days. On two occasions, we performed RAP induction on four consecutive days from the initial infection ( Figures 5C and H). From this preliminary screen, we were able to observe growth phenotypes ( Figures 5A and I). A minor growth phenotype was seen after KS of a protein containing a predicted RNA binding domain PBANKA_040140. Using KS, we have shown that this protein localised to a region around the nucleus in asexual stage parasites and appears to be important for normal parasite growth ( Figure 5A). A protein giving a severe growth phenotype was GAP50 (PBANKA_081900) ( Figure 5I). GAP50 is an integral part of the glideosome protein complex, which resides in the inner membrane complex (IMC) of apicomplexan parasites. It has recently been shown to have an important role in the morphological development of the related apicomplexan parasite *Toxoplasma gondii* ( Harding *et al.*, 2016; Harding & Meissner, 2014). GAP50 is expected to be an essential protein and we were able to confirm this predicted essentiality using KS. We confirmed the growth phenotype after GAP50 KS on numerous independent occasions and also in the independent parental background (KSP1), which gave a similar growth phenotype ( Figure S5D).

### KS phenotyping in sexual stages

GAP50 (PBANKA_081900) is expressed in both asexual and sexual stages. In addition to showing an asexual growth phenotype, we studied sexual stages. KS of GAP50 in gametocyte stages affected maturation to ookinetes, and although motile parasites were produced these were produced in much lower numbers and had an abnormal swollen morphology ( Figure 5J). We tested transmission through mosquitoes by treating GAP50 _KSP2_ infected mice with 1 mg/kg RAP 1 hour prior to feeding to mosquitoes. The number of midgut oocytes was counted between days 10 -14 and in three independent transmissions no GFP and mCherry positive oocysts were seen in mosquitoes fed on RAP-treated mice ( Figure 5J). On bite back to naïve mice, parasites were recovered from untreated mice with mean days to patency of 5 (N=3), whereas in the RAP-treated mice only on one occasion were parasites recovered and these were only observed 12 days after the bite back was performed. All parasites obtained from the untreated mice retained the expression of the GAP50::FKBP::GFP and the mCherry anchor ( Figure S9A), and were still responsive to RAP ( Figure S9B). The population resulting from the RAP-treated transmission contained parasites that had lost the FKBP::GFP tag on the GAP50. On further passage with rapamycin treatment a population was selected for which had WT (untagged) GAP50 as shown by absence of GFP fluorescence, and a Western blot showing the size of GAP50 consistent with the absence of the FKBP and GFP tag ( Figure S9C). This illustrates a strong selection pressure, suggesting that GAP50 is essential in *P. berghei* and demonstrates one possible, although rare, mechanism that disables the KS system.

## Discussion

We have demonstrated efficient and rapid, conditional relocalisation of proteins in *P. berghei* parasites. This is the first step in establishing KS as a useful tool for studying protein function in *Plasmodium spp*. In order to study a protein of interest (POI), the gene can be tagged using a simple single crossover homologous recombination to incorporate an FKBP::GFP tag onto the gene/protein in the KS parental background (currently KSP1 or 2). This requires one molecular cloning step to incorporate a homology region onto a plasmid containing the FKBP::GFP fusion along with a drug selection cassette. Following linearisation of the construct, well-established transfection technology will enable efficient integration of the tag onto the gene of interest. After drug selection (for ~ 1 week after transfection), the inclusion of the GFP allows for FACS enrichment ( Kenthirapalan *et al.*, 2012), generating an isogenic population of transfected parasites expressing the POI::FKBP::GFP fusion upon which further phenotypic characterisations can be performed. Preliminary experiments to confirm relocalisation were carried out by live microscopy on small samples of blood. We used transgenically expressed FKBP::GFP to show proof of principle of relocalisation technology in *Plasmodium*, and this line also represents a control line for subsequent protein specific experiments, showing that relocalisation of a relatively highly expressed transgenic protein (GFP) to the membrane anchor has no detrimental effect at any life cycle stage.

In previous studies, it has been shown that the relocalisation of POI through similar mechanisms abrogates its function, and therefore allows for the study of protein function ( Haruki *et al.*, 2008; Papanikou *et al.*, 2015; Robinson *et al.*, 2010). This allows this tool to have potentially broad ranging applications for protein function studies in *P. berghei*. However for some proteins, it is possible that POI function will not be eliminated by relocalisation and might require the generation of an alternative parental line in order to sequester the POI to a more discrete localisation (e.g. from the cytoplasm to the nucleus), for example to inactivate an enzyme by preventing it from being able to access its substrate. In other cases, the relocalisation may answer important questions about the importance of localisation for protein function. Possible examples would be the distinct localisation of many proteins in polarised life stages i.e. merozoites and ookinetes, where many organelles and proteins are localised to the apical end of the parasite ( Counihan *et al.*, 2013; Philip *et al.*, 2012) or the structural proteins, which may be essential for generation of this polarised morphology ( Harding *et al.*, 2016). Since the fluorescent proteins incorporated into the design of the system, immediate controls are possible, allowing the researcher to visualise the extent of relocalisation, and protein specific phenotyping assays can also make use of the fluorescent markers on both the POI and in the parental background. All of the above leads to a streamlined workflow to allow initial experiments to be performed without needing to perform time consuming experiments, such as western blots, as required with other inducible systems. Further advantages include significantly reduced animal usage and potentially allowing for a medium throughput approach to experimental design ( Figure S10).

We tested the KS system on a range of proteins designed to broadly test the system on proteins expressed at different life cycle stages and those with known (or predicted) functions, and mainly (to our knowledge) previously unstudied proteins. In this preliminary screen, we confirmed relocalisation is efficient on a wide range of *Plasmodium* proteins, which, due to the presence of the anchor on intracellular membrane structures, could include even nuclear proteins. While this parental line may be useful for screening a range of proteins, additional parental lines could be generated for relocalisation to more discrete localisations for specific questions. For example, we were able to show relocalisation of CITH protein, but preliminary results ( Figure S8B) showed we could generate apparently fully mature ookinetes despite significantly relocalised CITH in contrast to the previously published knockout ( Mair *et al.*, 2010) where no mature ookinetes were seen. This may be due to the still broad localisation of this protein at ookinete stages after rapamycin (RAP) relocalisation, and so further experiments will be needed to determine if protein localisation is essential for CITH function. Nonetheless in our small initial screen, we were able to identify two proteins (GAP50 and an RNA binding protein) with growth phenotypes after KS induction. KS of the IMC protein GAP50 resulted in a severe growth defect and was also used to demonstrate that the KS system can reveal phenotypes in both asexual and sexual stages. We observed that in the absence of RAP, the growth of all lines was comparable to the parental and to WT *P. berghei,* suggesting that the presence of the tag on the proteins tested was not detrimental.

Although there are several very recently established methodologies for the study of protein function in *Plasmodium* ( de Koning-Ward *et al.*, 2015), all come with some limitations and it is expected that no system will be efficient for every protein. One major advantage of KS over other systems is the speed at which protein relocalisation occurs. Complete relocalisation of GFP could be observed in live parasites as early as 1 minute after addition of RAP, making KS more rapid than even the most rapid of the other currently available knockdown systems, i.e. the auxin degron (15–60 minutes from induction of the system to maximal protein degradation ( de Koning-Ward *et al.*, 2015) ( Philip & Waters, 2015)). The speed of action makes KS-based technologies potentially ideal for dissecting dynamic developmental processes where protein inactivation on a scale of seconds gives a scale of temporal resolution previously unavailable. Another unique feature of the KS system is the ability to analyse KS efficacy at both a population and individual level. IFC is a technology that enables the analysis of large numbers of individual parasites within a population and has been shown to be a useful tool for the study of intracellular parasites, including recently the studies of invasion and size of *P. berghei* ( Bargieri *et al.*, 2013; Lin *et al.*, 2015). By using IFC, we have been able to show that all individuals within the population were responding to KS. This feature of the KS system relies on being able to resolve relocalisation by fluorescence microscopy, which may not always be possible. We show two examples of this in out screen, one protein with a subnuclear localisation in asexual stages, and one protein with a membrane localisation. More sensitive techniques for measuring displacement of protein are currently under development, which may allow for greater sensitivity for detecting relocalisation.

Importantly, KS functions *in vivo*. This is a feature of KS that is not yet practically possible in other protein level inactivation systems in *P. berghei* and means that KS will be a useful tool for studying many aspects of parasite-host interactions in an *in vivo* context. Re-localised proteins were detected in tail drop samples taken 20 minutes after IP injection of RAP. No relocalisation was observed in samples taken ≤15 min after IP injection, reflecting the time taken for RAP to enter the bloodstream. The FRB-RAP-FKBP complex is known to be very stable, and relocalisation was stable for at least 24 hours in blood stage parasites and was seen in ookinetes from mosquito midguts following transmission from an infected mouse treated with RAP. This meant that we were able to carry out preliminary growth curves over the course of a week to study the effect of protein KS on parasite growth and know that protein relocalisation (and potentially inactivation) was occurring very rapidly. Using the strong mCherry fluorescence of the parental line, along with Hoechst DNA stain and the GFP fluorescence on the tagged protein allows for highly informative growth analysis by flow cytometry, which potentially allows for determination of life cycle stages, as well as basic parasitaemia. Unbound RAP is less stable, and newly invaded parasites 24 hours after RAP did not show relocalisation. The rapid metabolism of RAP in mice also permits study of the invasive stages as they are transmitted back into mice. Pre-treatment of a mouse with RAP before feeding with infected mosquitoes affects KS in sporozoite stages, while the rapid metabolism of the drug means that emerging asexual blood stages exiting the hepatocyte, and which are not present until at least three days later, would not be affected by the drug. We can therefore expect to be able to perform KS on any protein of interest at a range of life cycle stages in order to determine protein function through the life cycle using a range of *in vivo* and *in vitro* assays.

In summary, we have developed a system that uniquely allows very rapid protein displacement in *P. berghei* both *in vitro* and *in vivo,* and which has the potential to be a very useful tool for the functional study of a wide-range of proteins in these important apicomplexan parasites.

## Methods

### Generation of plasmids

All plasmid manipulations were performed using traditional molecular biology methods with restriction enzymes purchased from NEB and other enzymes from Roche. Oligonucleotide primers were obtained from MWG-Biotech/Eurofins.

Plasmids used to generate the KS parental lines were created by modification of plasmid pG073 ( Sinha *et al.*, 2014) by addition of the FRB rapamycin dependent heterodimerisation partner domain ( Robinson *et al.*, 2010) and MCherry (red fluorescence; Clontech) domains fused to PbOTPT ( Mullin *et al.*, 2006) (PBANKA_110790; transmembrane domain protein) downstream of 1.6 kbp of HSP70 (PBANKA_071190) 5’ promoter/intergenic region to generate plasmid pG0089, which was used to generate the **K**nock- **S**ideways **P**arental line 1 (KSP1). This parasite line was negatively selected to generate line KSP1-M0 and cloned using conventional single cell cloning to generate line KSP1-m0cl2, which was used as a parental line for further transfections (referred to as KSP1 in the text). The plasmid pG0089 was then modified by replacement of the 3’ UTR region. The first ~500 bp of the p45/48 3’ UTR was excised with SmaI/AfeI and replaced with 492 bp of the translationally repressive 3’ UTR from P28 (PBANKA_051490) to generate plasmid PG0089-P28. This plasmid was digested with SacII prior to transfection into a WT *P. berghei* line to generate the parental parasite line KSP2. KSP2 was cloned to generate KSP2cl2, negatively selected to generate KSP2Cl2M1 and cloned again to generate KSP2cl2m1cl3 which was used as a parental line for further transfections (referred to as KSP2 in the text).

The construct used to express FKBP::GFP as a control (pG0111) was derived from pL0012 (Leiden Malaria Group, Leiden, The Netherlands) by addition of FKBP::GFP fusion generated by overlapping PCR to create plasmid pG0111. This was transfected into parasite lines KSP1 and KSP2 to generate lines GFP _KSP1_ and GFP _KSP2_, respectively, which were subsequently FACS sorted to generate isogenic lines for further experiments.

The construct used to tag proteins by single crossover with FKBP::GFP was derived from a modification of pL0031 (pL0031KH) by addition of the FKBP domain into the NcoI site in front of and in frame with GFP to generate plasmid pG0079. The FRB and FKBP domains were taken from plasmids, which were a gift from Margaret Robinson ( Robinson *et al.*, 2010), and amplified by primers to incorporate suitable restriction sites for cloning (all primers are detailed in Table S1). To generate tagged proteins of interest, primers were used, as detailed in Table SM1, to amplify approximately 1000 bp of the C terminal end of the gene of interest to exclude the stop codon. If no suitable unique restriction site for linearization was present either a KpnI or an EcoRV site was incorporated by overlapping PCR. All *P. berghei* sequences were amplified from *P. berghei* genomic DNA. *P. berghei* ANKA (HP) was obtained from Chris Janse at Leiden University Medical Centre and is also referred to as clone 15Cy1A. Forward and reverse primers for each gene of interest incorporated BamHI and NotI restriction sites, respectively, in order to clone into the NotI and BamHI sites in the MCS of pG0079 in frame with the FKBP::GFP.

All parasite lines were checked phenotypically (for appropriate fluorescence expression) and genotypically by PCR for presence of appropriately integrated constructs ( Figure S1).

### Parasite maintenance

*P. berghei* ANKA parasite, derived from line “HP” ( *P. berghei* ANKA HP was obtained from Chris Janse at Leiden University Medical Centre and was originally referred to as clone 15Cy1A), were maintained in female mice of TO or NIH strain (Harlan), weighing approximately 25 g. All animal work was performed in accordance with appropriate Home Office licensing. All efforts were made to ameliorate animal suffering, i.e: the strain of mice used was not susceptible to the severe complication of cerebral malaria and daily monitoring of parasitaemia was performed to ensure that parasitaemia never reached a level where suffering may occur. Blood was collected by cardiac puncture under terminal anaesthesia before suffering occurred. Experiments were performed using the minimum number of mice possible and the project was designed to result in a streamlined workflow in order to reduce animal usage (see Figure S10). For some experiments, mice were treated with 100 µl phenylhydrazine (PHZ; 12.5 mg/ml in physiological saline) by intraperitoneal (IP) injection 2 days prior to infection with *P. berghei*. Mice were infected with *P. berghei* by IP injection of 200 µl of cryopreserved stocks, or by intravenous (IV) injection of 200 µl of purified schizont stage parasites. Sulfadiazene at 30 mg/litre was provided in drinking water, when required, to obtain pure gametocyte populations. Daily monitoring of parasitaemia was performed by analysis of methanol-fixed Giemsa stained thin smears of blood obtained from a small tail drop.

### Parasite culture methods

Mouse blood that contained parasites at 3–10% parasitaemia was collected via cardiac puncture and placed into pre-warmed (37°C) schizont culture medium (RPMI1640 containing 25 mM HEPES, 5 mM hypoxanthine, 20% FCS, 10 mM sodium bicarbonate, 100 U/ml penicillin and 100 µg/ml streptomycin). Parasites were cultured to schizont stage at 0.5% haematocrit in schizont culture media in a sealed flask gassed with 5% O _2_/5% CO _2_ at 37°C with shaking at 40 rpm for up to 27 hours. Schizonts were enriched by flotation on a 55% Nycodenz ^®^ density gradient. To obtain pure sexual stages, a PHZ-treated mouse with an infection at 3–7% parasitaemia was treated with sulfadiazene (30 mg/litre in drinking water) for two days to kill asexual stages, then blood was collected by cardiac puncture. Ookinete stage parasites were obtained by inoculating blood containing mature gametocytes at 2% haematocrit in ookinete culture medium (RPMI1640 containing 25 mM HEPES, 5 mM hypoxanthine, 20% FCS, 10 mM sodium bicarbonate, 100 µM xanthurenic acid at pH 7.6) at 21°C. Mature ookinetes were analysed after 20–24 hours.

### Parasite transfection methods

Parasites were transfected at schizont stage by electroporation using Amaxa nucleofector machine (Lonza), as previously described ( Philip *et al.*, 2013). One mouse provided sufficient schizonts for up to 8 transfections. A suspension of Nycodenz-enriched schizonts was resuspended in Nucleofector II (Lonza) and mixed with 5 µg of purified linearised plasmid DNA. After electroporation, parasites were immediately injected into a tail vein (IV) of a mouse. In total, 24 hours post-infection, parasites were placed on positive selection by administration of pyrimethamine in drinking water (70 μg/ml).

### Parasite cloning methods

For conventional cloning, infected blood containing parasites at 0.2 - 0.4% parasitaemia was diluted in schizont media, such that there was 0.4 parasites per 200 µl of media. A total of 200 µl was injected (IV) per mouse into 10 mice. This yielded 3 or 4 positive mice per experiment and these were deemed to be clonal. To recycle the selectable drug marker in cloned populations of KSP1 and KSP2, negative selection was carried out by administration of 5-flurocytosine (Sigma) 1mg/ml in drinking water ( Orr *et al.*, 2012). For FACS “cloning”, parasites were cultured to early schizont stage and placed in FACS buffer (see below). FACS cloning was performed on a BD FACSAria I cell sorter equipped with 405 nm, 488 nm and 640 nm lasers and using a 70 µM nozzle. Parasites positive for GFP were sorted for high purity into 100 µl of rich PBS in a 0.5 µl microcentrifuge tube and diluted so that 50 parasites could be injected (IV) into the tail vein of one mouse, in order to generate an isogenic population.

### Induction of KS

To visualise KS relocalisation by microscopy, a tail drop of blood was taken and incubated in 500 µl rich PBS with 5 µM Hoechst and with or without rapamycin. Rapamycin (RAP; Sigma) from a stock in DMSO was diluted to a final concentration of 200 nM, unless otherwise stated. Incubations were generally for 15–30 minutes at 37°C. Samples were centrifuged at 10,000 rpm, resuspended in 50 µl of supernatant, a 6 µl drop was placed onto a glass microscopy slide covered with a 22×22 mm cover slip and sealed with nail varnish for live microscopy, as described below. To allow us to identify emerged gametes, in some cases parasites were counterstained with Ter119 to identify the red blood cell membrane. Parasites were incubated in 500 µl of a 1:500 dilution of Ter119-alexa350 (ebioscience) in rich PBS for 20 minutes at 37°C, with or without addition of 200 nM RAP (Sigma). Parasites were then centrifuged in an Eppendorf centrifuge at 10,000 rpm for 30 seconds and placed into 500 µl ookinete media to induce activation or rich PBS for the unactivated sample. After briefly vortexing samples were centrifuged again as before and resuspended in 50 µl of supernatant. 6 µl of sample was placed on a microscope slide and covered with a 22×22mm coverslip then sealed with nail varnish for live microscopy. To induce KS in cultures, parasites were placed into schizont culture media with or without the addition of RAP at 200 nM, unless otherwise stated. DMSO alone had no effect on parasite cultures compared to no treatment. RAP at this concentration, and up to 1000 nM, had no effect on WT parasite growth or development. Parasite cultures were visually inspected after overnight growth in all concentrations of RAP tested and no obvious difference in growth rate or quality of maturation were observed. For *in vivo* induction of KS, RAP stock at 4 mg/ml was diluted 6.25 µl into 200 µl of PBS for IP injection per mouse to dose at 1 mg/kg, unless otherwise stated. Mice were assumed to weigh 25g. IV injection of equivalent amount of DMSO had no effect on parasite growth or KS ( Figure S5). The number of mice used for each experiment was between 2 mice for initial phenotyping of a line up to 8 mice for initial control drug concentration testing. Usually 4 mice were used per line for growth analysis.

### Flow cytometry analysis

Parasites were analysed by flow cytometry on either an LSRII (BD) flow cytometer equipped with 405 nm, 488 nm, 561 nm and 640 nm lasers or a CyAn-ADP (Beckman Coulter) flow cytometer equipped with 405 nm, 488 nm and 640 nm lasers. For experimental analysis, parasites from a tail drop of blood (~2 µl) or 0.5 ml of pelleted culture was resuspended in 0.5 ml of rich-PBS (rich PBS: PBS (Roche) with 20 mM HEPES, 20 mM glucose, 4 mM NaHCO _3_, 0.1% BSA) containing Hoechst at 5 µM and incubated in the dark for 15–30 minutes at 37°C. After staining, parasites were spun at 10,000 rpm for 30 seconds and resuspended in 1 ml FACS buffer (FACS buffer: PBS (Roche) with 2 mM HEPES, 2 mM Glucose, 0.4 mM NaHCO _3_, 0.01% BSA, 2.5 mM EDTA) and filtered through a 40 µm pore nitex® membrane (Cadisch Precision Meshes). Samples were run on the flow cytometer at a rate of around 10,000 events per second, and for most experiments 500,000 events (RBC excluding debris) were acquired. Uninfected red blood cells subject to the same staining procedures were also run, as well as non-fluorescent control parasites when required to define gating strategies. Analysis was performed using Kaluza software (version 1.3), Beckman Coulter), and compensation settings and gating strategies were based on appropriate single expressing control parasites.

### Imaging flow cytometry analysis (IFC)

Samples were prepared for IFC analysis by magnetically enriching for parasites from 100 – 200 µl of infected blood using a LD500 magnetic column (Miltenyi) and custom built magnet. After washing in rich PBS, parasites were resuspended in 50 µl IFC buffer (dPBS with 0.1% BSA) at a concentration of ~1X10^7 parasites per ml. Samples were filtered through a 40 µm pore nitex ^®^ membrane before analysis. In total, 5,000–50,000 parasites (or a 10 minute time course) were acquired on an ImageStream ^X®^ MkII analyser (Amnis-Merck) equipped with 405,488,561 and 632 nm lasers. Single control line parasites KSP2 and GFP _KSWT_ were acquired in the same way and used for compensation controls. Analysis (including compensation) was carried out using IDEAS software (version 6.2; Merck). After gating on GFP positive cells, focussed cells were identified in the mCherry channel using the gradient RMS, and the colocalisation feature in IDEAS was used to identify colocalisation in GFP and mCherry channels. For time course analysis, RAP was added to prepared parasites, samples was vortexed to mix and placed immediately on the analyser. Acquisition commenced 60 – 68 seconds after addition of RAP and was continued for 10 minutes. Gated populations containing parasites acquired from 0–100, 100–200, 200–400 and 400–600 seconds were analysed for colocalisation. In order to compare this time course to untreated samples, which were run separately, a merged file was generated for analysis and untreated samples were identified based on time and object number. Further details are in Figure S5 and Supplementary methods.

### Microscopy

Live cell and fixed cell immunofluorescence microscopy was performed on a Deltavision Core microscope using an Olympus 100X/1.40objective, a CoolSNAP_HQ2/HQ2-ICX285 camera and softWoRx software (Version: 5.5.1 Release 3). A Z-spacing of 0.15 µm was used and images were deconvoluted using softWoRx conservative ratio setting with medium filtering. Images presented show a single slice (or a maximum projection if indicated) and were further processed to generate figures using ImageJ/FIJI software (version 2.0; NIH). All microscopy was performed on multiple independent occasions with similar results. Some live cell imaging was performed on an Axioplan II (Zeiss) microscope using a 100X objective and processed using Volocity (version 4.1; Improvision) and Image J software.

### Mosquito transmissions

Parasites were grown in pairs of untreated mice until parasitaemia reached 2–5%. One mouse was treated by IP injection of 1 mg/kg RAP 30 minutes before being anaesthetised. In total, 30 minutes after administration of anaesthetic, mice were placed onto a cage of 250 6–8 day old unfed *Anopheles stephensi* mosquitoes. Mosquitoes were allowed to feed for 12 minutes in dark conditions at 21°C. To analyse midgut ookinete phenotype, five midguts were dissected from fed mosquitoes 24 hours after feeding. Midguts were placed into 500 µl of rich-PBS, disrupted by passage (3 times) through a fine gauge (insulin) needle, and debris removed by centrifugation at 500 x g for 20 seconds. Blood from the midgut was diluted in rich PBS and placed on a LD500 column on a custom built magnet to isolate ookinetes. These were eluted away from the magnet in rich-PBS, concentrated by centrifugation in a microcentrifuge at 10,00 rpm for 1 minute, stained with Hoechst (5 µM) before being placed on a microscope slide for imaging on a Deltavision core fluorescent microscope. Alternatively unstained parasites were analysed by IFC on an ImageStream ^X^ mkII imaging flow cytometer, as described above. Between days 7 and 14 midgut oocysts were counted on isolated midguts using a Leica M205FA stereo fluorescence microscope equipped with a 1X and 5X objective and filter sets suitable for GFP and mCherry. The number of fluorescent oocysts per midgut for ~ 15 mosquitoes per experiment were counted. Transmission “bite back” experiments were performed on day 18. Naïve mice were anaesthetised and infected mosquitoes (usually ~25 per experiment) were allowed to feed for 8–12 minutes. To minimise suffering the mice were anaesthetised appropriately for the minimum time possible. Eyes were protected from light and mice were kept warm throughout the process with a cotton wool blanket and transferred to a heated cage for recovery. Sensitive areas (i.e. nose and paws) were protected from bites. After recovery, mice were monitored for parasitaemia from day 4 to day 14. Any resulting parasites were analysed for fluorescence expression and response to KS induction.

## Data availability

Raw parasitaemia counts for growth curves in this manuscript (relating to Figure 4, Figure 5 and Figure S4) have been uploaded to the online data repository OSF: doi, 10.17605/OSF.IO/SBBKD ( Hughes, 2017) ( https://osf.io/sbbkd).

## Supplementary material

**Figure S1. Knocksideways parental lines.** ( **A**) Schematic of constructs used to generate the KSP1 parental lines for KSP1 (upper) and KSP2 (lower). Constructs are identical except for the 3’ UTR, which is a constitutive P45/48 UTR for KSP1 and gives a higher expression level than the repressive P28 UTR in KSP2 (lower). Plasmids were generated by conventional molecular cloning methods and linearized with sacII restriction enzyme (S) before transfection into wild type Pb-ANKA parasites. ( **B**) PCR results showing integration of the FRB::mCherry::PboTPT (also referred to as FRB::mCherry::TM) construct into the 230p locus. Left panel primers across the integration region (outside integration homology arm), expected product size is 2894bp in WT. Middle panel linking HSP70 promoter within the construct to 230p locus (upstream of integration targets), expected product size is 4104 bp before negative selection and 1380bp after negative selection. Right panel primers linking part of the construct including mCherry and PboTPT to 230p locus (downstream of integration targets), expected product size 2368 bp. Size markers are 1kb+ ladder (Invitrogen). ( **C**) Histogram of mCherry fluorescence measured by flow cytometry on an LSRII cytometer showing a parasite line with no mCherry fluorescence (507, grey), KSP1 with a constitutive 3’ UTR (green) and KSP2 with a repressive 3’ UTR (red). The brightest parasites in the original KSP1 line are gametocytes. The apparent absence of this population in the line with the repressive 3’ UTR is due to a reduction in fluorescence to similar levels to the main population and not due to absence of gametocytes (confirmed by microscopy, e.g. Figures S1F and 2B). The histogram shows infected red blood cells only gated on a positive DNA stain. ( **D**) KSP2 parasites show a greatly increased ookinete conversion rate compared to KSP1. ( **E**) Membrane localisation of FRB::mCherry::TM is conferred by the PboTPT protein fusion, as a parasite line expressing FRB::mCherry alone shows diffuse mCherry localisation in the parasite cytoplasm. Upper image shows mCherry and Hoechst merge, lower shows DIC image, scale bar = 5 µm. ( **F**) Images of KSP1 (left) and KSP2 (right) at trophozoite, schizont, gametocyte and ookinete stages, showing similar FRB::mCherry localisation. Very few ookinetes were ever observed in the KSP1 line. In KSP2 line ookinetes internal membranous structures are visible (arrow).

**Figure S2. Knocksideways in an independent parental background.** Images of GFP relocalisation in line GFP _KSP1_, which was generated by transfection of a construct to express FKBP::GFP into the parental line KSP1 that expresses FRB::mCherry::TM with an HSP70 5’ UTR and P45/48 3’ UTR. Results seen were identical to that observed in the GFP _KSP2_ control line as described in the main text. Images of GFP _KSP1_ relocalisation after rapamycin (RAP) treatment in asexual ( **A**) and gametocyte stages ( **B**). Asexual parasites were counterstained for 15 minutes in 5 µm Hoechst with or without 200 nm RAP before live imaging. Sexual stage parasites were stained with the red blood cell membrane stain Ter119 for 15 minutes with or without 200 nm RAP before being placed into activation buffer (ookinete culture media) and live imaging. ( **C**) Images of relocalisation of GFP after *in vivo* exposure to RAP. Samples of blood from a tail drop were taken 20 minutes after IP injection of 1 mg/kg RAP per mouse and showed GFP relocalisation. ( **D**) Images of parasites after ~20 minutes RAP exposure *in vitro* at concentrations from 1.6 nm to 1000 nm. ( **E**) Images of a parasite taken 3 minutes after addition of 200 nM rapamycin *in vitro.* Scale bar = 5 µm. Images are representative of >5 parasites observed per condition on at least 2 independent occasions. See also IFC results in Figure 3 and Figure S6.

**Figure S3. GFP Knocksideways induction *in vivo*.** ( **A**) Images of live GFP _KSP1_ parasites from tail drop samples taken 4h (upper) and 24h (lower) after IP injection of 0.6 mg/kg rapamycin (RAP). Showing a young asexual trophozoite stage and a mature gametocyte stage parasite. Scale bar = 5 µm. Representative image from >10 parasites observed on 2 independent occasions. ( **B**) Images of live GFP _KSP2_ parasites taken after a second dose of 0.5 mg/kg RAP.

**Figure S4. Images of GFP knocksideways mosquito midguts.** Images of an intact midgut (upper) and a cropped image of a single oocyst (lower) taken from a mosquito fed on a mouse infected with GFP _KSP2_ and treated with 1 mg/kg RAP 1 hour prior to the feed. GFP in ookinetes isolated from midguts from the same experiment 24 hours after feed showed colocalisation of GFP with mCherry ( Figure 4F) but oocysts show no colocalisation. Oocyst images were taken 6 days after feeding. Dissections were performed and images taken on a Leica stereo fluorescence microscope. Representative of 6 midguts in two independent experiments.

**Figure S5. GFP knocksideways images and growth curves.** ( **A**) Images of GFP _KSWT_ line with FKBP::GFP in a WT parental background without (upper) or with (lower) 200 nM rapamycin (RAP). Growth curve shows % parasitaemia with IP administration of 1 mg/kg RAP on day 2 and 3 (arrow) (N=2 +/-SD). ( **B**) Images of KSP2 parental line without (upper) or with (lower) 200 nM RAP and growth curve of KSP2 with addition by IP of 1 mg/kg RAP on day 3 and 4 (arrow) N=3 +/- SD. ( **C**) Parasites recovered from bite back experiments with RAP treatment during transmission at gametocyte/ookinete and sporozoite stage retained expression of GFP and mCherry and were still responsive to RAP. Representative images of >10 parasites observed in two independent experiments. ( **D**) Images of GFP _KSP1_ control line without (upper) or with (lower) 200 nM RAP. Left growth curve shows GFP _KSP1_ with addition by IP of 0.5 mg/kg RAP on day 2, 3 and 4, and right growth curve shows GAP50 _KSP1_ growth curve without or with (red line) addition of RAP at 0.5 mg/kg on day 2,3 and 4 (arrows). Minus rap mice were treated by IP injection of an equivalent amount of DMSO in PBS as a vehicle control. GFP positive parasitaemia was counted and of individual growth curves are shown.

**Figure S6. Knocksideways relocalisation of GFP occurs at low rapamycin (RAP) concentrations.** Imaging flow cytometry analysis of parasites. Panels show a histogram of colocalisation between GFP and mCherry for – RAP (blue) and plus RAP (red) at indicated concentration, untreated parasites were acquired separately and files merged for analysis. The colocalisation feature wizard was used in IDEAS software and bright intensity colocalisation score for each image is calculated. A representative gallery of images for each drug concentration is shown, scale bar = 7 µm. The non-RAP treated sample is the same for each analysis. The table shows the number of parasites included in the analysis, % colocalisation and median colocalisation score for each treatment. Representative of two independent experiments. See also Figure S2.

**Figure S7. Schematic of gating strategy for imaging flow cytometry time course.** See also Supplementary methods and Figure 3. After acquisition of events gated on size *vs* aspect ratio to collect only single RBCs, the illustrated gating strategy was used collecting ( **A**) bright red/green parasites, ( **B**) parasites in focus in the mCherry channel. The colocalisation wizard in IDEAS software was used, then results plotted for time separated regions ( **C** and **D**). To define smaller time windows a smaller gate was drawn on the time plot ( **E** and **F**).

**Figure S8. Further characterisation of *Plasmodium berghei* protein knocksideways.** ( **A**) Images of activated PBANKA_101390 parasites minus and plus 200 nM rapamycin (RAP) *in vitro*. Gametocytes were stained with the RBC membrane marker Ter119 then activated to trigger emergence of the gametocyte from the host RBC. Images show the GFP signal remains with the parasite and is not associated with the host membrane (see also Figure 4). ( **B**) Images of CITH PBANKA_130130 KS after RAP addition (200 nM) at gametocyte stage and 24 h maturation to ookinete. Morphologically mature ookinetes were observed in some cases despite significant displacement of the CITH protein. Scale bar = 5 µm. Images representative of >6 ookinetes per experiment observed in three independent experiments.

**Figure S9. Analysis of GAP50 _KSP2_ transmitted lines.** ( **A**) Flow cytometry plots showing GAP50 _KSP2_ cultured schizonts from the line prior to transmission (top), after transmission bite back (TBB) with no rapamycin (RAP) (middle) and after transmission with RAP treatment at gametocyte stage (lower). Mature schizonts as gated on high mCherry and high Hoechst (DNA content) (left plot) were analysed for GFP expression (Right plot). Numbers show the % of mature schizonts that express GFP (GAP50::FKBP::GFP). ( **B**) Transmitted parasite lines (as described in ( **A**)) show a growth phenotype after a single dose of RAP. Parasite infections at 2–4% parasitaemia (upper plots) were treated with 1 mg/kg RAP (IP) and effect on parasitaemia monitored by flow cytometry the following day (lower plots). Numbers show the % parasitaemia based on mCherry and Hoescht stain. ( **C**) Parasite lines were passaged into a naïve mouse and maintained without (-) or with (+) respectively two further doses of 1 mg/kg rapamycin. Resultant populations had lost the GFP fluorescence measured by flow cytometry analysis of schizonts ( **C**), and a western blot of parasite lysate probed with an antibody raised against GAP50 show that the population expresses only WT size GAP50 as compared to the increased size when the GAP0::FKBP::GFP fusion protein is expressed ( **D**).

**Figure S10. Suggested workflow for initial knocksideways (KS) analysis.** Workflow and predicted time required for initial analysis of KS.

**Figure S11. Effect of rapamycin (RAP) on wild type parasites.** Plots of male activation centres after treatment with RAP.

**Supplementary methods. Imaging flow cytometry (IFC) methods for IFC time course**.

**Table S1. Primers used in knocksideways experiments.**
