# Supplementary figures and images for: Rapid inducible protein displacement in Plasmodium in vivo and in vitro using knocksideways technology
Source: Wellcome Open Res. 2017 Mar 14;2:18. [Version 1] doi: 10.12688/wellcomeopenres.11005.1 (PMC5395084; doi:10.12688/wellcomeopenres.11005.1)

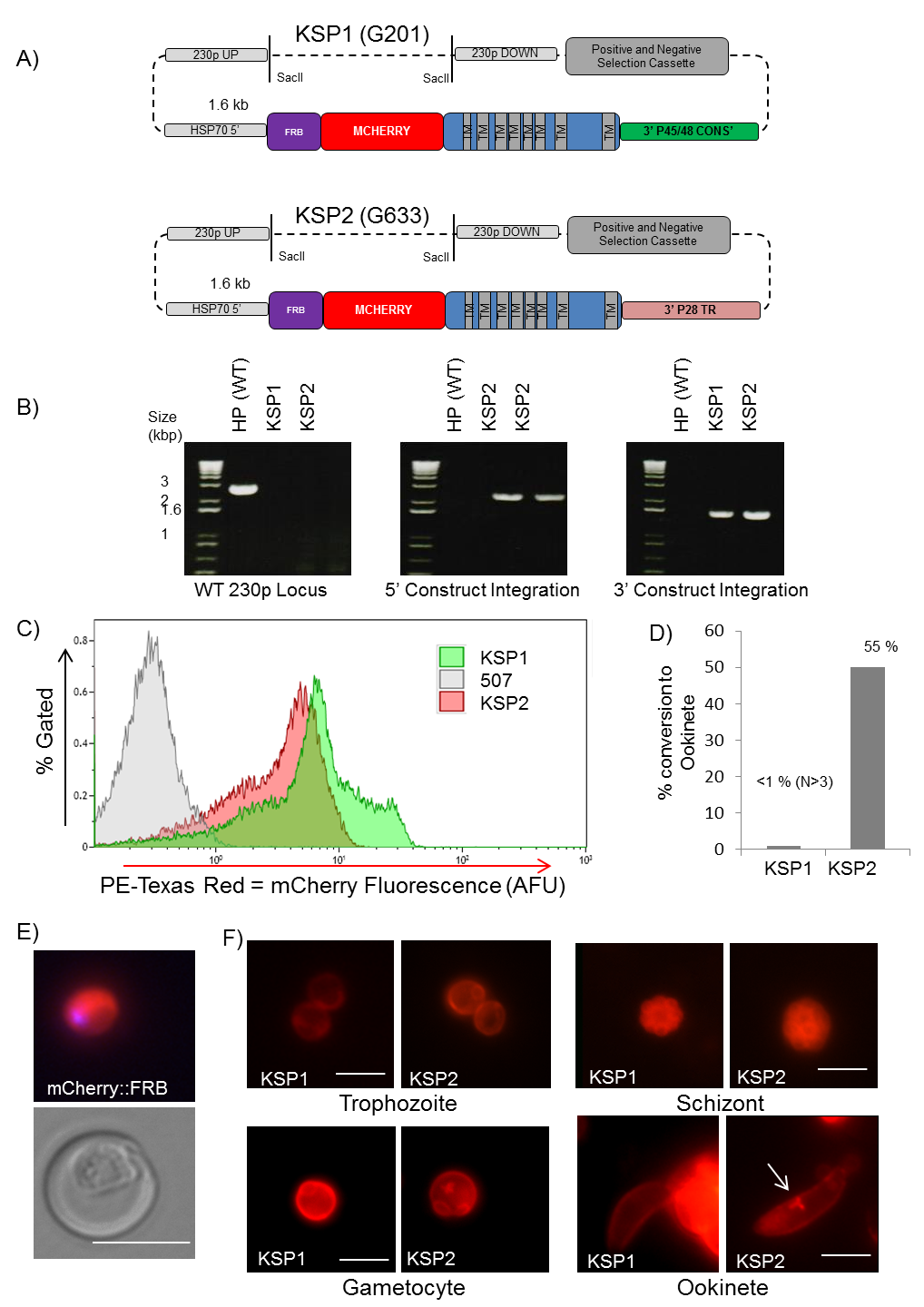

Supplement: Supplementary file 1 [file wellcomeopenres-2-11865-s0000.tgz › 186882db-b0be-4c6c-bd49-2ff81d33d5c1.tif]

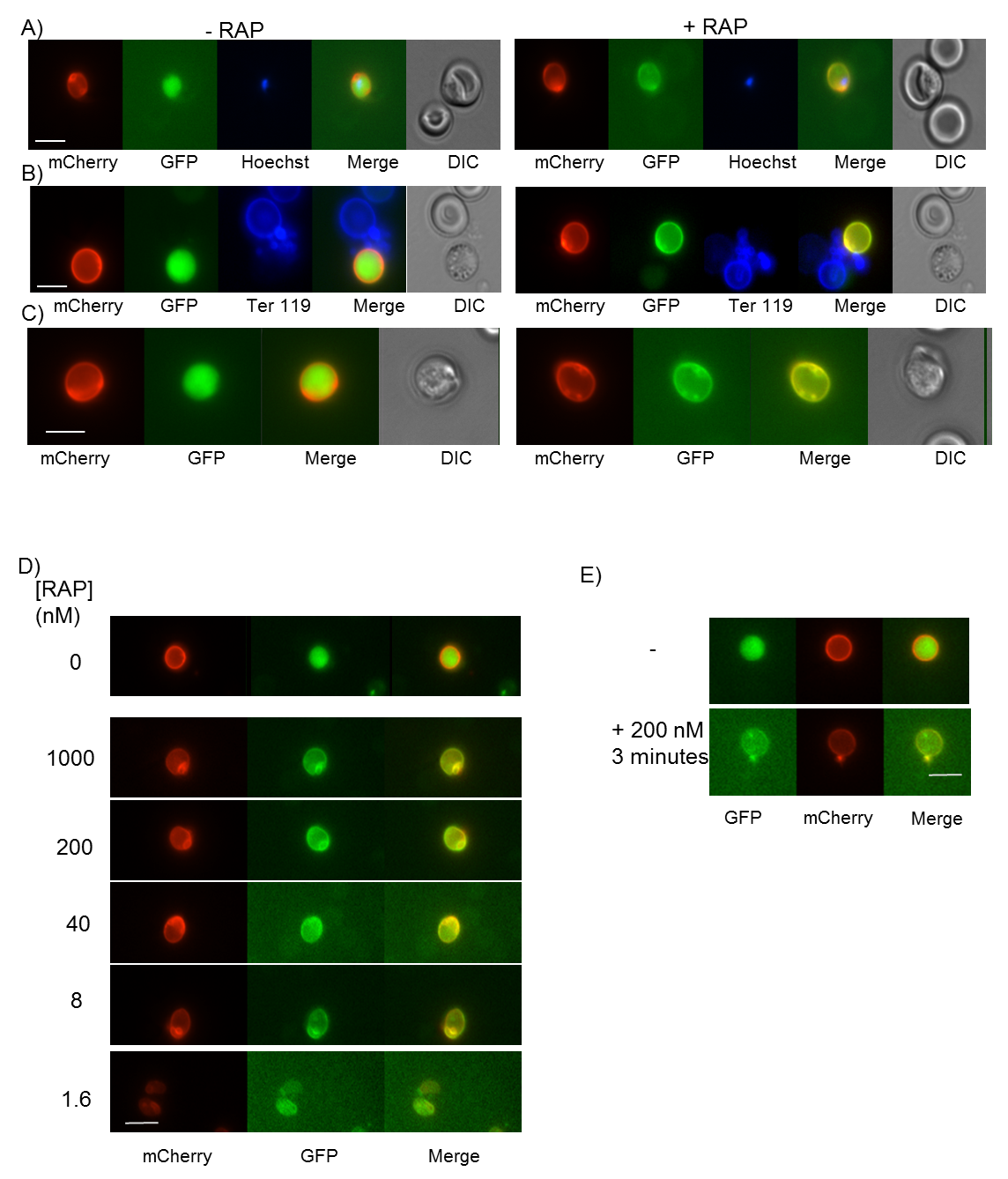

Supplement: Supplementary file 2 [file wellcomeopenres-2-11865-s0001.tgz › 737b7883-54b1-49da-8672-0354c52a224b.tif]

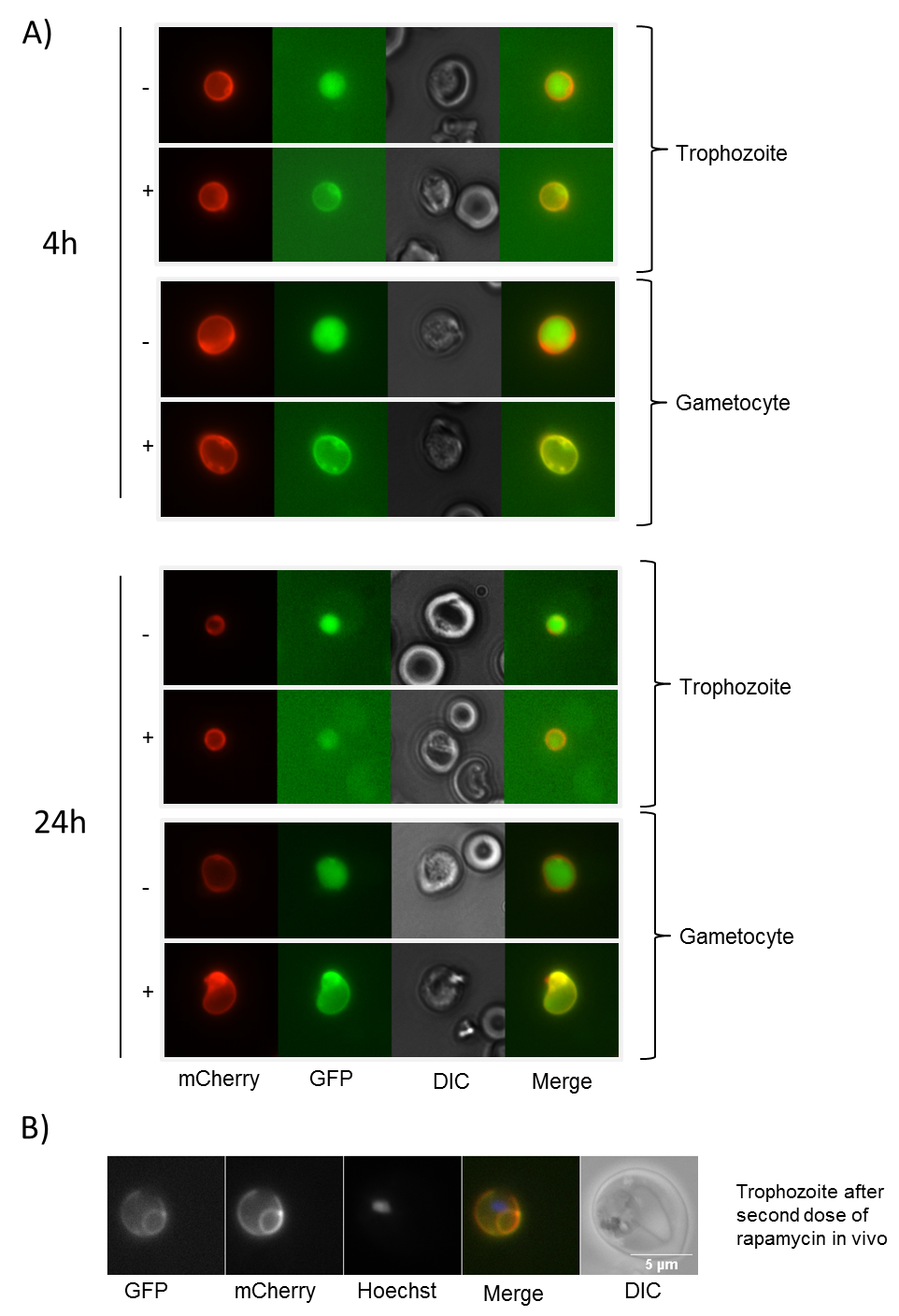

Supplement: Supplementary file 3 [file wellcomeopenres-2-11865-s0002.tgz › 5ccf141e-5641-4642-b107-57d2b5c5e560.tif]

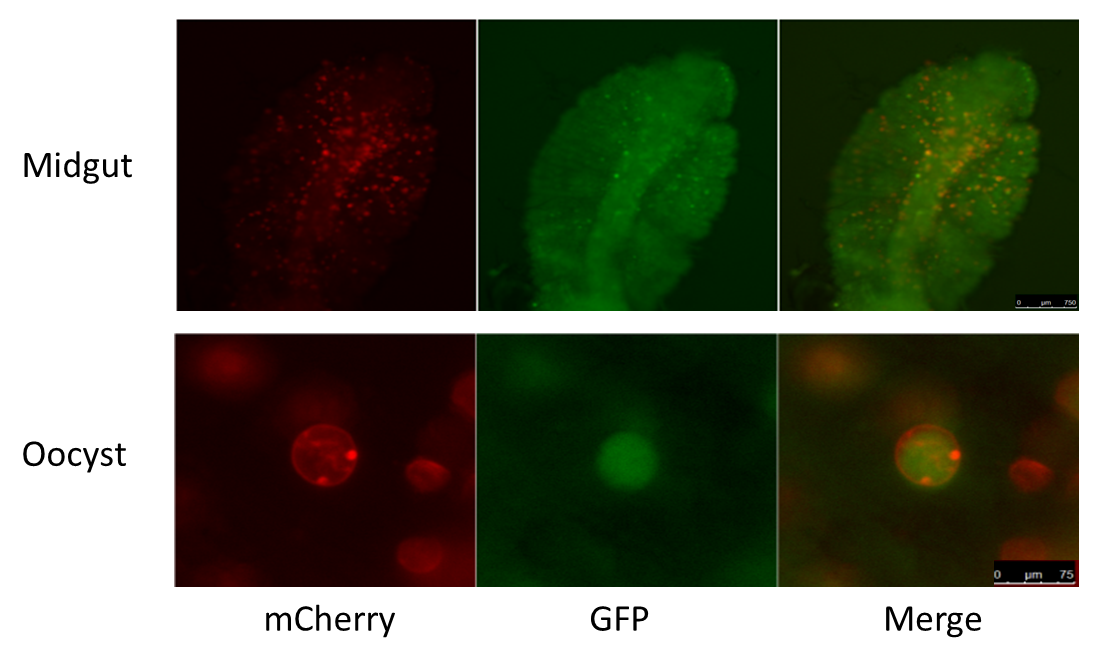

Supplement: Supplementary file 4 [file wellcomeopenres-2-11865-s0003.tgz › 00731b1e-2255-4fcf-ba2b-e6b2096b7522.tif]

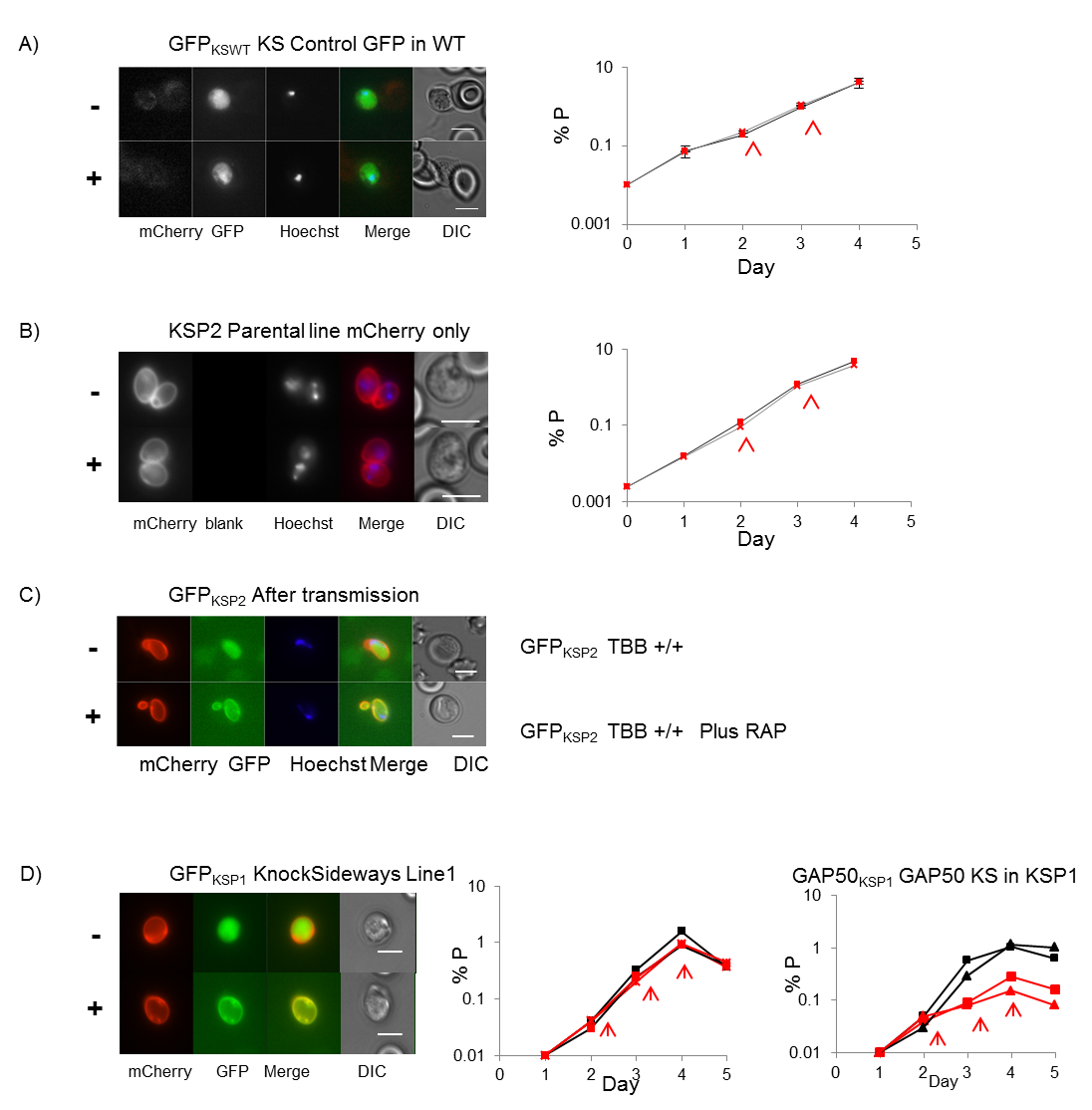

Supplement: Supplementary file 5 [file wellcomeopenres-2-11865-s0004.tgz › 31adf95c-7a02-4949-8fde-019e1197ec57.tif]

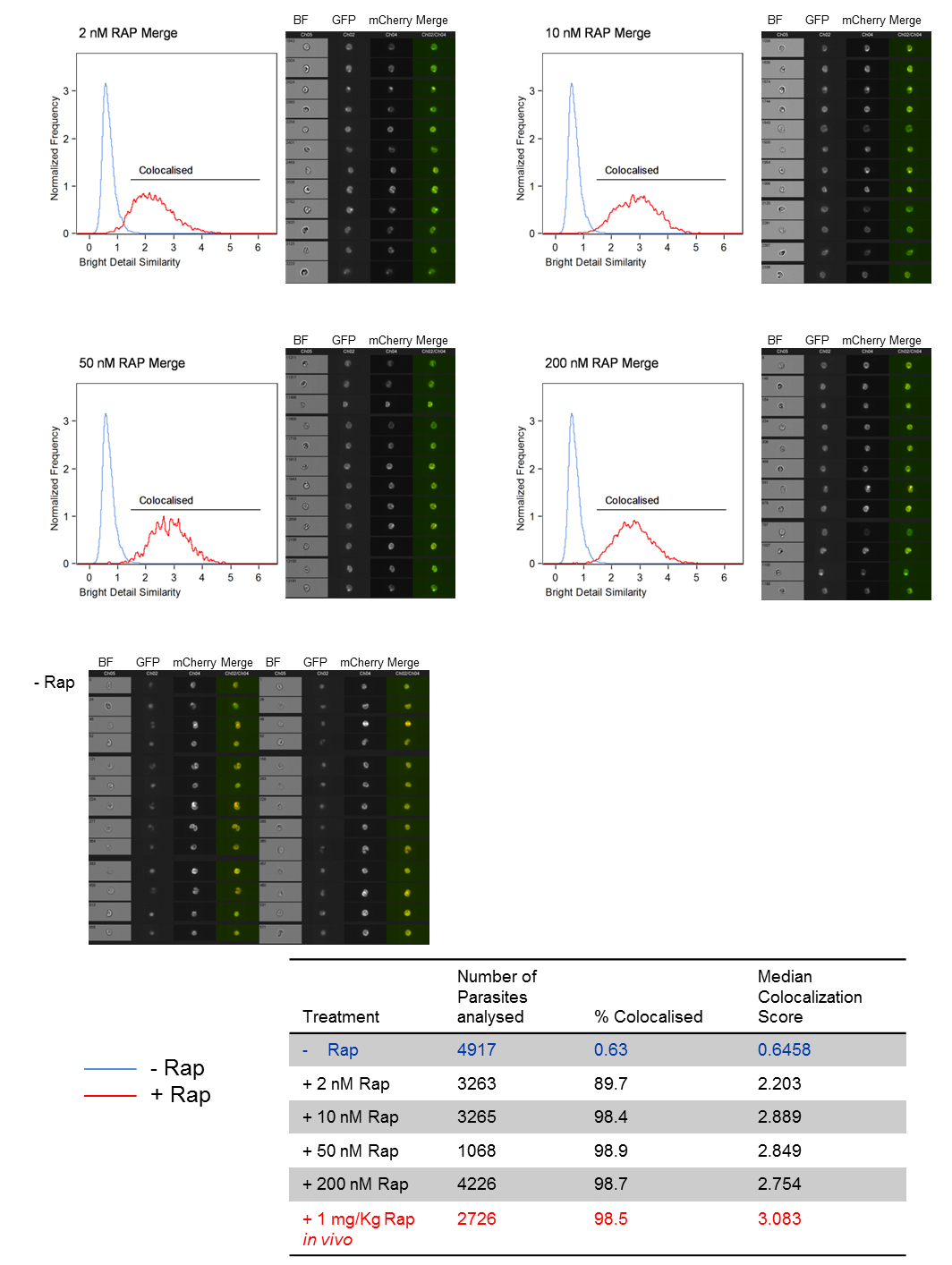

Supplement: Supplementary file 6 [file wellcomeopenres-2-11865-s0005.tgz › fc03d893-7434-4e31-9e19-32b5f11a967b.tif]

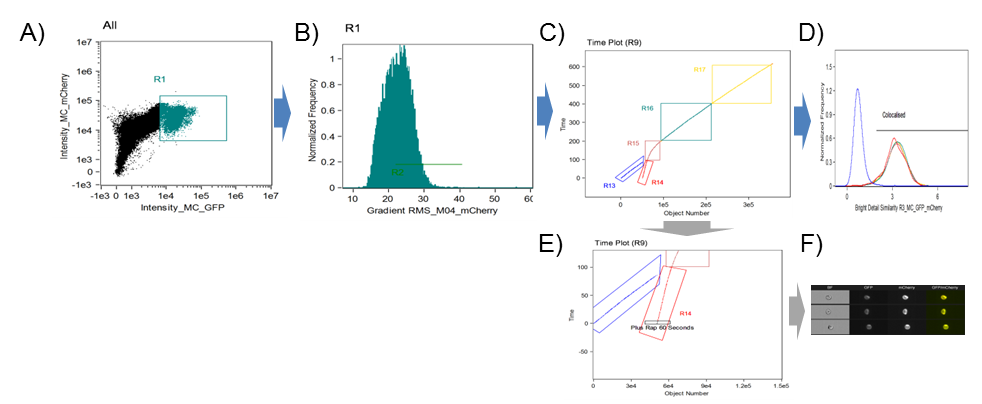

Supplement: Supplementary file 7 [file wellcomeopenres-2-11865-s0006.tgz › b0b5c9b5-f0c5-4f94-988c-8958c1ba4401.tif]

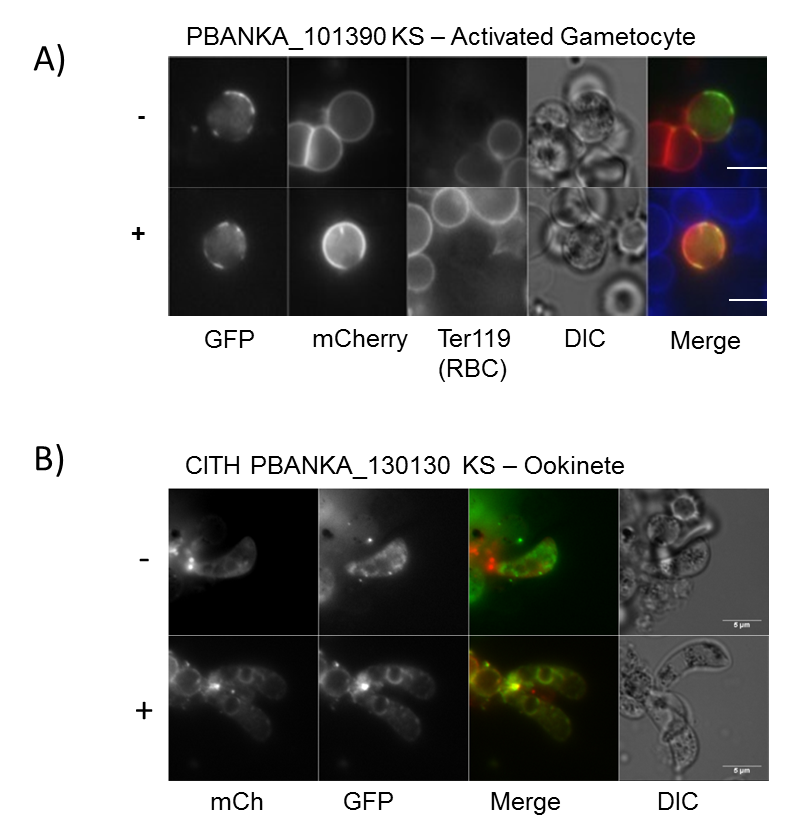

Supplement: Supplementary file 8 [file wellcomeopenres-2-11865-s0007.tgz › 30d508c5-88e2-43f1-8d46-d88313b40007.tif]

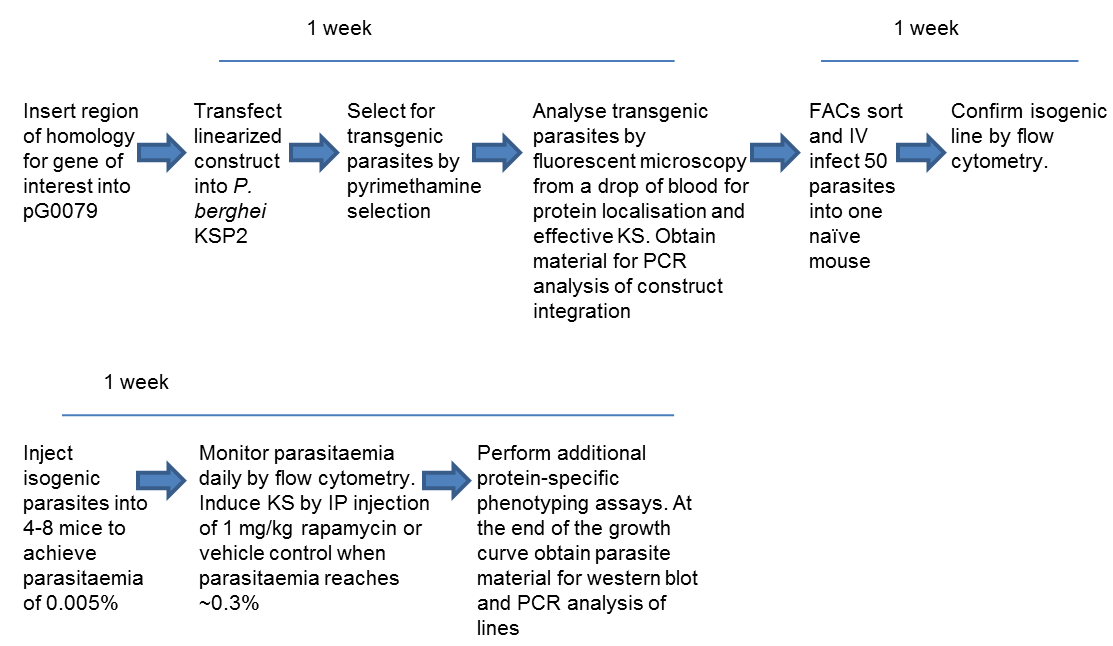

Supplement: Supplementary file 10 [file wellcomeopenres-2-11865-s0009.tgz › e166d416-9443-41a3-ae94-63d67cd65555.tif]

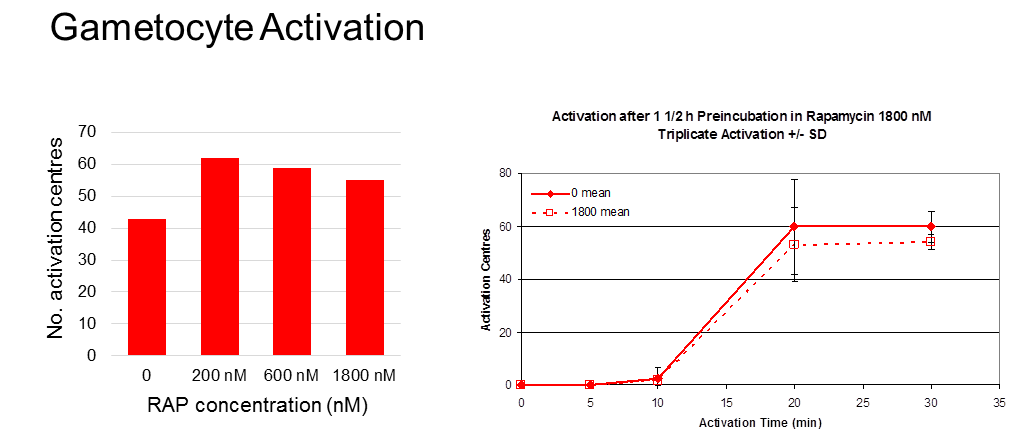

Supplement: Supplementary file 11 [file wellcomeopenres-2-11865-s0010.tgz › 80292101-a86e-4f8d-861b-c95326e9c0bc.tif]
