# Supplementary material for: Rapid inducible protein displacement in Plasmodium in vivo and in vitro using knocksideways technology
Source: Wellcome Open Res. 2017 Mar 14;2:18. [Version 1] doi: 10.12688/wellcomeopenres.11005.1 (PMC5395084; doi:10.12688/wellcomeopenres.11005.1)
Supplement: Supplementary file 12 [file wellcomeopenres-2-11865-s0011.tgz › b5846613-3e6e-43c9-8c78-15ac7a815bba.docx]

**Supplementary Methods**

**Imaging flow cytometry (IFC) methods for IFC time course**

**SAMPLE PREP**

1. In total, 200 µl of blood containing mixed blood stage parasite of line GFP_KSP2_, was magnetically enriched using an LD column. KSP2 and GFP_WT_ (100 µl each) was also enriched in the same way to provide single colour (mCherry/GFP respectively) controls. Each control sample was resuspended in 100 µl of ImageStream FACS buffer and filtered through 40 µm pore Nitex. GFP_KSP2_ parasites were resuspended in 200 µl and split into two samples of 100 µl each.

**SAMPLE ACQUISITION**

1. In total, 5000 events of GFP_WT_ parasites were acquired with the 488 nm and 561 nm lasers set to 200 mA after ensuring that at this power no pixels were saturated. No brightfield or SSC laser illumination was on to enable these to be used as compensation controls.
2. In total, 5000 events of KSP2 parasites were acquired with the 488 nm and 561 nm lasers set to 200 mA and no brightfield or SSC laser illumination
3. In total, 50,000 events of untreated enriched GFP_KSP2_ parasites (sample 1) were acquired the 488 nm and 561 nm lasers set to 200 mA, brightfield and SSC laser illumination on.
4. A total of 1 µl of 20 µM rapamycin was added to 100 µl of enriched GFP_KSP2_ parasites (sample2), the sample was vortexed for 2 seconds then immediately loaded onto the machine and run with the same settings as before.
5. Acquisition was commenced as soon as possible, this was 60-68 seconds after addition of rapamycin (RAP) and continued for 10 minutes

**SAMPLE ANALYSIS**

1. A compensation matrix was created in the IDEAS analysis software using single colour controls
2. The minus RAP sample and the RAP time course files were merged and compensation applied.
3. Samples were gated sequentially on:
   1. Area *vs* aspect ratio of brightfield to collect RBCs (performed during acquisition)
   2. GFP *vs* mCherry to collect GFP and mCherry positive parasites
   3. Gradient RMS on mCherry to collect parasites with the mCherry membrane in focus
   4. The Colocalisation Wizard feature was run on the above population for colocalisation between channel 2 (GFP) and channel 4 (mCherry).
   5. A plot of Object number vs Time of the above population was generated and gates drawn to define the untreated sample (R13) and time segments of the rapamycin treated sample. Statistics for colocalisation were obtained and plotted for each gated population.
