# Supplementary material for: Rapid inducible protein displacement in Plasmodium in vivo and in vitro using knocksideways technology
Source: Wellcome Open Res. 2017 Mar 14;2:18. [Version 1] doi: 10.12688/wellcomeopenres.11005.1 (PMC5395084; doi:10.12688/wellcomeopenres.11005.1)
Supplement: Peer review history

# Peer review: Referee response for version 1

**DOI:** 10.21956/wellcomeopenres.11865.r21811

**Published:** 2017-04-12

**Authors:** Geoffrey I. McFadden

**Affiliations:** 1 School of BioSciences, University of Melbourne, Parkville, VIC, Australia

The manuscript reports the application of an inducible reverse genetic tool to study protein function in *Plasmodium berghei*, a rodent model for malaria. Reverse genetics in *Plasmodium* parasites has enjoyed limited success thus far for two reasons: no one-size-fits-all inducible knockout or knockdown system has yet emerged, which makes deletion of essential genes very difficult; and transformation can only be done at blood stage, which makes manipulations across the life cycle awkward to nay impossible for many genes.  To date, no particular conditional gene modification tool has emerged as the obvious protocol of choice.  Thus, further additions to this toolkit, particularly relatively straightforward ones with apparently wide utility, are a welcome addition. This contribution is therefore of very strong value. The authors have generated two options for parental lines. With these other researchers should be able to tag their protein of interest in these parental lines and perform a knock sideways experiment with modest effort. Importantly, they can likely perform this experiment at various points in the parasite life cycle— *in vivo* in mice and perhaps mosquitoes, but also *in vitro* in blood and sexual stages, and perhaps in *in vitro* cultured liver stages. Such versatility is not available in other systems, so this tool is particularly worthwhile. Other inducible knock down systems have proven difficult to apply across the life cycle, particularly with inherent lags between induction and outcome. The rapidity with which the protein of interest can be displaced is thus a very distinct advantage— particularly for ephemeral stages like zygotes and ookinetes—and all kinds of valuable phenotyping scenarios can be contemplated.

 A key shortcoming of this knock sideways technology, which could be discussed briefly, is that it seems unlikely (as presently constructed) to work for proteins sequestered into compartments such as the apicoplast, mitochondrion, nucleus, endomembrane system [ER/Golgi/food vacuole], the parasitophorous vacuole, or the so-called PEXEL proteins exported to the red cell. Indeed, looking at figure 5D for protein PBANKA_030530 it appears that the nucleolar protein is not displaced at all. In theory, creating different sink locations in other parental lines could open up the possibility of displacing these compartmentalised proteins, but that is perhaps a discussion for another day. Perhaps the authors would like to flag these options for future development of the tool.

 Another unexplored limitation/advantage is whether the FKBP domain can be used as an N-terminal fusion as an alternative to the currently reported C-terminal fusions. Again, this is something to explore in future and not at all necessary for addition to this first report of efficacy, but the authors might like to point out that there is no conceivable reason why N-terminal tagged proteins couldn't be knocked sideways.

 One thing that can be clarified is the location of the sink protein (the *P. berghei* homologue of the plastid phosphate translocator known as *Pf*oTPT. Mullin *et al. *showed that this multimembrane pass protein resides in the outer membrane of the apicoplast, and this targeting was maintained with either N or C-terminal tagging with triple hemagglutinin epitopes [1]. However, it seems that adding the mCherry and FRB domains on the N-terminus has drastically perturbed this targeting, with essentially all of the sink protein localising to the parasite plasma membrane. This is likely because the tyrosine residue at position 10 in *Pf*oTPT (position 9 in the *Pb* homologue) is essential to apicoplast targeting [2] and appending the sink domains in front of it destroys its context. Happily the N-terminus still apparently projects into the cytosol because the displacement clearly works. Did the authors intend for the sink to be on the outer apicoplast membrane? If so, was this intended to better facilitate KS, and why? I imagine that putting the sink domains at the C-terminus of *Pb*oTPT would result in it being targeted to the apicoplast if that is the desired location, but again I don't think this is required for the current communication.

 Some minor fixes:

- On page 2 in the figure legend the species designation component for *Sac*II needs to be italicised.
- On page 10 it should read 'our' not 'out'

I have read this submission. I believe that I have an appropriate level of expertise to confirm that it is of an acceptable scientific standard.

## References

[1] MullinKALimLRalphSASpurckTPHandmanEMcFaddenGI: Membrane transporters in the relict plastid of malaria parasites. *Proc Natl Acad Sci U S A*.2006;103(25) : 10.1073/pnas.0602293103 9572-7 10.1073/pnas.0602293103 16760253PMC1480448

[2] LimLSayersCPGoodmanCDMcFaddenGI: Targeting of a Transporter to the Outer Apicoplast Membrane in the Human Malaria Parasite Plasmodium falciparum. *PLoS One*.2016;11(7) : 10.1371/journal.pone.0159603 e0159603 10.1371/journal.pone.0159603 27442138PMC4956234

# Peer review: Referee response for version 1

**DOI:** 10.21956/wellcomeopenres.11865.r21136

**Published:** 2017-04-05

**Authors:** Volker T. Heussler

**Affiliations:** 1 Institute of Cell Biology, University of Bern, Bern, Switzerland

The paper by Hughes and Water describe a new very useful tool to investigate protein function in *P. berghei*. The authors adapted the inducible knocksideway technology for *P. berghei* and show convincingly that it works for a number of proteins. The paper is well written and the data fully support their conclusions. I would like to particularly point out the quantitative imaging approach analyzing co-localization in thousands of cells.

 I have no major concerns but I would like to ask the authors to discuss the limitations of the method in more depth. Although the method has been proven useful for *in vivo* blood stage parasites, most likely it would be of limited use for *in vivo* liver stage parasites because rapamycin, in the concentration used, might have very strong effects of parasite liver load as observed in mice treated with similar concentrations (1mg/kg vs. 2,5 mg/kg) (Prado *et al.* 2015). This could make the interpretation of the results very difficult. Another point that should be addressed more clearly is that the technology might not be useful for proteins that are secreted or transported into certain cell compartments. Finally, it would be good to discuss the possibility to also use the KS technology in the mosquito to study oocyst development by feeding rapamycin to the mosquitoes infected with the corresponding transgenic parasites.

 Other comments:

- The authors state the FKBP-FRP complex is very stable in presence of rapamycin. How long does this association last if the drug is removed?
- Please add size bars to the images in Figure 2E
- Please increase the size of the parasites in Figure 3, 4C, 4F and 5. In the printed version the parasites in these images are hardly visible.

I have read this submission. I believe that I have an appropriate level of expertise to confirm that it is of an acceptable scientific standard.

## References

[1] PradoMEickelNDe NizMHeitmannAAgop-NersesianCWackerRSchmuckli-MaurerJCaldelariRJanseCJKhanSMMayJMeyerCGHeusslerVT: Long-term live imaging reveals cytosolic immune responses of host hepatocytes against Plasmodium infection and parasite escape mechanisms. *Autophagy*.2015;11(9) : 10.1080/15548627.2015.1067361 1561-79 10.1080/15548627.2015.1067361 26208778PMC4590598

# Peer review: Referee response for version 1

**DOI:** 10.21956/wellcomeopenres.11865.r21103

**Published:** 2017-03-30

**Authors:** Avnish Patel

**Affiliations:** 1 London School of Hygiene & Tropical Medicine, Faculty of Infectious and Tropical Diseases, London, UK

Huges and Waters demonstrate the robust application of knocksideways technology to the rodent parasite *P. berghei.*

 This method of allows tentative protein function to be interrogated by conditionally sequestering the endogenous protein in a non-native cellular localisation. Whilst a variety of conditional systems exist that allow gene product knockdown or knockout, these methodologies can have a significant lag time in efficacy. This can lead to complications in phenotypic interpretations in which secondary effects may occur during the slow depletion of the gene target. Knocksideways has initially been demonstrated to show rapid kinetics and efficacy in mammalian cell types allowing protein sequestration in a timescale of minutes, which mitigates secondary effects. Huges and Waters apply this technology to *P. berghei *demonstrating rapid kinetics of efficacy *in vitro* and importantly *in vivo* on multiple life cycle stages.

 Overall the study presented is sound and the results justify the conclusions. However, some minor points require clarification and modification.

 Whilst the authors have demonstrated the rapid kinetics of association between the membrane anchor and protein of interest, they have not looked in detail at the kinetics of reversibility of the rapamycin induced. The data looking at protein association or lack thereof in oocysts formed from rap treated gametocytes gives some indication of this suggesting that the interaction is not long lived i.e. on a scale of days and the loss of association is most likely due to turnover of rap associated complexes in this time frame. It may be of interest to include data in which *in vitro* parasites are rap treated to allow complex formation and subsequently loss of association is monitored after rap wash out, granted that this may not be achievable *in vivo*.

 Some of the knocksideways images of parasite proteins are difficult to interpret. For example, PBANKA_093250 is said to re-localise to the periphery of developing merozoites however, in the images presented here only one merozoite structure shows this to some degree of prominence (bottom left hand merozoite). It would be beneficial to include some line profiles of +/- Rap treatment images, as was done for the GFP control in figure 2E, to allow a clearer interpretation of the results.

 Whilst the authors have mentioned that the CITH knocksideways results do not match the results obtained from the CITH knockout, they suggest that CITH is functional when re-localised. This assumes that re-localisation is complete and it maybe that all of the protein is not fully sequestered which is difficult to tell from co-localisation alone. Following on from this overall suitability of KS to study protein function could be discussed. It may be worth illustrating the suitability of KS to certain gene targets such as enzymes, as re-localisation of these proteins could cause non-native effects due to activity at the site of re-localisation.

 Some minor spelling and figure labeling:

 The panel in Figure 5B is shown with coloured fluorescence however the other panels are not, is this for clarity? Worth mentioning in the figure legend.

 Supplementary Figure S1B middle panel is labelled KSP2 twice.

 Stated in the discussion ‘We show two examples of this in out screen, one protein’ maybe should read ‘in our screen’.

I have read this submission. I believe that I have an appropriate level of expertise to confirm that it is of an acceptable scientific standard.

# Peer review: Referee response for version 1

**DOI:** 10.21956/wellcomeopenres.11865.r21135

**Published:** 2017-03-29

**Authors:** Julian C. Rayner

**Affiliations:** 1 Malaria Programme, Wellcome Trust Sanger Institute, Hinxton, UK

Experimental genetics has had a major impact on our understanding of *Plasmodium *parasite biology (elegantly reviewed in PMID 25978707) - deleting genes of interest has led to significant insights since the first knockout lines were published twenty years ago (PMID: 9108483 and 9002517). A central problem with the field has always been the question of how to tackle genes that are essential for blood stage growth, which is the stage at which experimental genetic manipulations are carried out. If a gene is essential for blood stage growth, by definition it can not be deleted; this is because *Plasmodium *parasites are haploid during blood stage development, so deleting the only copy of an essential gene will kill the parasite before it can be selected for. However, it is the function of these essential genes that are in many cases the most interesting to study. This central conundrum has led to the development and application of numerous conditional technologies to *Plasmodium *parasites, allowing functional study of essential genes by disrupting function at the DNA (recombinases such as Cre-loxP, FLP-frt or DiCre), RNA (Tet regulatable transactivators, glmS riboswitch to mediate mRNA degradation) or protein (DD destabilisation domain); PMID 25978707 is a comprehensive source of references for these technologies. All of these conditional approaches have their strengths and weaknesses, and while several success stories have been published for each approach, many more attempts that have failed almost certainly sit unpublished in the lab books of PhD students and postdocs around the world. There is as yet no clear one size fits all strategy that means that no further conditional systems are required.

 This paper adds a new approach, knocksideways, to this expanding conditional genetic toolbox. Used initially to study protein trafficking in mammalian cells, the system relies on rapamycin mediated dimerisation of two protein fragments, one of which is tagged to the protein of interest by genetic manipulation, and the other membrane anchored to a “sink” site such as the plasma membrane. Addition of the small molecule induces dimerisation of the two fragments, and hence mis-localisation of the tagged protein to the sink site, potentially ablating function, depending on how context-dependent the function of the tagged protein is.

 In the paper, the authors use the rodent *P. berghei *model system, which has the twin advantages of being highly genetically tractable and allowing genetic interrogation across the entire life-cycle of the parasite. A proof of principle is developed using fluorescent reporters, and imaging and flow cytometry confirms that relocalisation is efficient and rapid. Utilising the advantages of *P. berghei*, the system is validated *in vivo *by treating infected mice with rapamycin, and in transmission stages by analysing midguts from infected mosquitoes. Ten endogenous *P. berghei *genes were then tagged and the effect of relocalisation on blood-stage growth investigated, along with in one case, the effect on transmission stages.

 Overall the study is technically sound and clearly demonstrates the broad applicability and utility of the knocksideways approach in *P. berghei*, forming a complement to the recent publication of a similar proof of principle which tagged 12 targets in *P. falciparum *(PMID: 28288121). The quantification of relocalisation using both confocal microscopy and imaging flow cytometry is a particular strength - colocalisation scores are generated for >6,000 parasites using the latter approach, which lends a robustness to the colocalisation data that is usually lacking in *Plasmodium *imaging studies. Validation of the system *in vivo *and through mosquito stages is also a significant step, and provides a clear opportunity for work that is not easily accessible in *P. falciparum*, where mosquito infections are much more challenging.

 Technical points that could do with additional clarification include:

1. Localisation of the anchor appeared different in blood stages and ookinetes, when apical localisation was more apparent in some images (Figure 4D particularly), and also varied somewhat even within blood stages in the multiple strains shown in Figure 5. Co-localisation post-rapamycin treatment was also less compelling in ookinetes, at least as judged by imaging flow cytometry. Has the relative abundance of the anchor between intracellular compartments been quantitated at different stages, and does it differ consistently between lines? Could this affect relocalisation efficiency?
2. The +rap effect seems to wear off by the time oocysts develop - are there any speculative methods to dose infected mosquitoes with rapamycin?
3. One key question is whether mislocalisation is sufficient, in the majority of cases, to impede function. In the 10 proteins studied in Figure 5, one is very clearly expected to be essential, GAP50. Knock sideways of GAP50 clearly has a growth phenotype (Fig 5I), but is this as extensive as would be predicted from complete ablation of an essential protein? Is it possible to calculate the extent of functional reduction based on predicted growth curves for 50% killing, 80% killing, etc?
4. Some comment on the likely scalability and strengths/weaknesses would have been interesting. There are clearly strengths - simple genetic manipulation, rapid mislocalisation. However, a potential weakness is the extent to which mislocalisation ablates function, which will of course be target dependent. Testing on known essential genes, whose products are targeted to different organelles, would be a useful next step.

 

 In summary, a paper with high technical standards that comprehensively introduces a new conditional approach to the *Plasmodium *genetic toolkit that has very specific advantages of rapidity and applicability to transmission stages.

I have read this submission. I believe that I have an appropriate level of expertise to confirm that it is of an acceptable scientific standard.

# Peer review: Referee response for version 1

**DOI:** 10.21956/wellcomeopenres.11865.r20994

**Published:** 2017-03-27

**Authors:** Tobias Spielmann

**Affiliations:** 1 Department of Parasitology, Bernhard Nocht Institute for Tropical Medicine, Hamburg, 20359, Germany

In this paper Hughes and Waters established the knock sideways technique to study protein function in *Plasmodium berghei*. The KS technique is based on ligand induced heterodimerisation of two domains (FKBP and FRB) and can be used to rapidly remove a protein of interest from its site of action, thereby inactivating it. The power of this conditional protein inactivating method has already been demonstrated in other organisms. As so far no ubiquitously applicable conditional gene or protein inactivation system exists for malaria parasites, this technique adds an important further tool for the study of essential proteins in this parasite. A particular attribute of KS is its speed to inactivate the target which has been shown to avoid compensatory effects that can arise with other gene and protein inactivating approaches (Robinson *et al.*, Dev Cell 2010).

 To establish this system in *P. berghei*, Huges & Waters first generated parasite lines stably expressing what they term the 'sink' (the part of the heterodimerisation system that sequesters away the protein of interest). For this they use an apicoplast outer membrane protein (that however in most cases was found at the plasma membrane). They carefully evaluate these cell lines and use GFP fused to the second part of the heterodimerisation system to demonstrate rapid and ligand dependent recruitment of GFP to the sink in different parasite stages (including both mouse and mosquito stages). They then go on to test the system with a series of *P. berghei* proteins, several of which have not previously been analysed. Growth assays showed that two of these proteins (one of them the IMC protein GAP50) are needed for efficient parasite growth. For GAP50 an Ookinete phenotype was also shown, demonstrating the use of KS to study essential proteins in transmission stages.

 The strength of the conditional protein inactivation system presented in this paper is its speed and its applicability *in vivo*, permitting functional studies in both the mouse and the mosquito. The only small concern I have is that although this paper demonstrates the general applicability of the system in *P. berghei*, the efficiency of mislocalisation was somewhat unclear for some of the analysed proteins. Maybe there would be room to further improve the mislocaliser (i.e. the sink). While this is clearly beyond the scope of this paper, it may be a point worth discussing. The authors state that in most cell lines the mislocalisation appeared to be complete and occurred in all visualised parasites. While not practical for all of the targets shown in this paper, it would be nice to have some measure of the efficiency of mislocalisation for some of the proteins where mislocalisation did not seem to be complete (for instance for PBANKA_040140 and PBANKA_093250).

 The protein with the strongest phenotype, GAP50, may be the most problematic in this respect, as its location at the IMC often is very close to the parasite plasma membrane, which is also the site it is mislocalised to after addition of rapamycin (this is particularly obvious in the ookinete images). While in this case the phenotype indicates successful mislocalisation, this may become an issue with other targets for which no phenotype is observed and it then is unclear whether failure to mislocalise or dispensability of the target is the reason for this. The authors note the possibility to use other mislocalisers and this may solve this problem in the future.

 Additional small points:

1. Some of the labels in Figure 5 carry an asterisk but this is not explained in the legend.
2. I would encourage the authors to find a way to increase the size of their microscopy images in the figures (especially Figure 5, but also Figure 4 and others). The details of the images are difficult to see in a regular print out of the paper. There is some dead space that could be used to rearrange the panels to make possible larger images.

Overall this is an important addition to the tool box to study essential malaria proteins. Some quantification of the mislocalisation levels for a couple of the cell lines could further improve the paper and would be nice to have. Finally, if possible, the size of the microscopy images should be increased.

I have read this submission. I believe that I have an appropriate level of expertise to confirm that it is of an acceptable scientific standard.
